# Transcriptional analysis of *South African cassava mosaic virus*-infected susceptible and tolerant landraces of cassava highlights differences in resistance, basal defense and cell wall associated genes during infection

**DOI:** 10.1186/1471-2164-15-1006

**Published:** 2014-11-20

**Authors:** Farhahna Allie, Erica J Pierce, Michal J Okoniewski, Chrissie Rey

**Affiliations:** School of Molecular and Cell Biology, University of the Witwatersrand, 1 Jan Smuts Ave, Braamfontein, Johannesburg, 2000 South Africa; Functional Genomics Center, Zurich, UNI ETH Zurich, Winterthurerstrasse 190, CH-8057 Zurich, Switzerland

**Keywords:** Transcriptome profiling, Cassava, Next-generation sequencing, Geminivirus, South African cassava mosaic virus, Tolerance, Susceptibility

## Abstract

**Background:**

Cassava mosaic disease is caused by several distinct geminivirus species, including *South African cassava mosaic virus*-[South Africa:99] (SACMV). To date, there is limited gene regulation information on viral stress responses in cassava, and global transcriptome profiling in SACMV-infected cassava represents an important step towards understanding natural host responses to plant geminiviruses.

**Results:**

A RNA-seq time course (12, 32 and 67 dpi) study, monitoring gene expression in SACMV-challenged susceptible (T200) and tolerant (TME3) cassava landraces, was performed using the Applied Biosystems (ABI) SOLiD next-generation sequencing platform. The multiplexed paired end sequencing run produced a total of 523 MB and 693 MB of paired-end reads for SACMV-infected susceptible and tolerant cDNA libraries, respectively. Of these, approximately 50.7% of the T200 reads and 55.06% of TME3 reads mapped to the cassava reference genome available in phytozome. Using a log_2_ fold cut-off (p <0.05), comparative analysis between the six normalized cDNA libraries showed that 4181 and 1008 transcripts in total were differentially expressed in T200 and TME3, respectively, across 12, 32 and 67 days post infection, compared to mock-inoculated. The number of responsive transcripts increased dramatically from 12 to 32 dpi in both cultivars, but in contrast, in T200 the levels did not change significantly at 67 dpi, while in TME3 they declined. GOslim functional groups illustrated that differentially expressed genes in T200 and TME3 were overrepresented in the cellular component category for stress-related genes, plasma membrane and nucleus. Alterations in the expression of other interesting genes such as transcription factors, resistance (R) genes, and histone/DNA methylation-associated genes, were observed. KEGG pathway analysis uncovered important altered metabolic pathways, including phenylpropanoid biosynthesis, sucrose and starch metabolism, and plant hormone signalling.

**Conclusions:**

Molecular mechanisms for TME3 tolerance are proposed, and differences in patterns and levels of transcriptome profiling between T200 and TME3 with susceptible and tolerant phenotypes, respectively, support the hypothesis that viruses rearrange their molecular interactions in adapting to hosts with different genetic backgrounds.

**Electronic supplementary material:**

The online version of this article (doi:10.1186/1471-2164-15-1006) contains supplementary material, which is available to authorized users.

## Background

Cassava, *Manihot esculenta* Crantz, is a tropical crop that is important for food security and income generation for many poor farmers in several Asian and African countries. Fresh tubers of cassava are suitable for consumption by both humans and animals, and provide the most important dietary source of calories for more than a billion people in about 105 countries, providing an estimated one third of calorie intake [1]. Cassava’s tolerance to unfavourable conditions and abiotic stress make it an excellent crop, in comparison with other cereals such as wheat, rice and maize, for small-scale farmers with limited resources. [2, 3]. Cassava starch is being exploited for its numerous industrial applications, including bioethanol, processing for the paper industry, pellets for animal feed, and thickeners in the food industry [4].

Cassava mosaic disease (CMD) is the most important biotic constraint of cassava production in sub-Saharan Africa [5, 6]. CMD is caused by whitefly-transmitted viruses of the genus *Begomovirus* (family *Geminiviridae*), including *South African cassava mosaic virus*-[South Africa:99] [NCBI-AF155806] (SACMV) [7]. SACMV has two circular DNA molecules, designated DNA-A and DNA-B, of approximately 2.8 kb, both of which are required for systemic infection of plants. Six genes are encoded by DNA-A, whereas two genes are encoded by DNA-B. DNA-A viral strand encodes for the coat protein (CP) (AV1 ORF), and AV2 which functions as a suppressor of host RNA silencing, thereby modulating symptoms, or may also be involved in host specificity. The minus strand of DNA-A has four open reading frames (ORFs) that encode for the Rep associated protein (AC1), a transcriptional activator (TrAP/AC2), a replication enhancer (Ren/AC3), and the AC4 protein. The AC4 ORF lies entirely embedded within the coding region of the Rep protein, and it is the least conserved of all the geminiviral proteins, both in sequence and in function [8].

In past years there have been high levels of resistance/tolerance to CMD found in several Nigerian cassava landraces including TME3 [9–11]. By using classical genetic techniques such as genetic mapping, resistance in several cassava cultivars was thought to be attributed to the presence of a major dominant resistance (R) gene, namely CMD2 [10, 11]. Furthermore, several molecular markers have been associated with CMD2, including SSRY28, NS158 and RME1 [10]. Currently, further efforts are being made in order to dissect the genetic architecture of cassava resistance and other economically important traits using an EST-derived SNP and SSR genetic linkage map approach [12]. However, more recently, in addition to the activation of effector triggered immunity by R genes, host RNA silencing has been identified as a major antiviral defence mechanism [13]. Viruses can both induce and target RNA silencing, and have evolved a number of strategies to overcome RNA-silencing mediated host defence mechanisms via their multifunctional proteins, some of which can act as suppressors of RNA silencing (VSR), and which are also able to interfere with host miRNA pathways leading to disease induction and symptoms [reviewed in 13]. Viral genome methylation has also been shown to be an epigenetic defence against DNA geminiviruses [14]. Plants use methylation as a defence against DNA viruses, which geminviruses counter by inhibiting global methylation. In a study with *Beet curly top virus* (BCTV) in *Arabidopsis* plants, tissue recovered from infection showed hypermethylated BCTV DNA, and AGO4 was required for recovery [14]. Symptom remission or ‘recovery’ is a phenomenon reported in several plant studies, including pepper infected with the geminivirus, *Pepper golden mosaic virus* (PepGMV) [15], and has been associated with TGS and post-transcriptional gene silencing (PTGS) mechanisms [16].

Plants have developed both highly specialized defence responses to prevent and limit disease. Many disease responses are activated locally at the site of infection, and can spread systemically when a plant is under pathogen attack [17–20]. This initial response is usually termed basal or broad immunity which may be sufficient to combat the viral pathogen, or may lead to further specific resistant responses, namely induced resistance, often triggered by specific recognition and interaction between virus and host resistance proteins encoded by R genes [21–23]. This defence activation may be to the detriment of the plant, as fitness costs may often outweigh the benefits, because energy and resources are redirected toward defence, and normal cellular processes such as growth and yield are affected [24]. In many cases, in the absence of a speedy, effective and persistent basal immune response, plants will be susceptible, unless virus-specific R genes are present in that plant species/cultivar/variety. In order to minimise fitness costs, signalling molecules and pathways coordinating pathogen-specific defences are activated. Signalling molecules are predominantly regulated by salicyclic acid (SA), jasmonic acid (JA), and ethylene (ET) pathways which are known to act synergistically or antagonistically with each other in order to minimise fitness costs. Specific induced resistance is usually associated with direct pathogen recognition, resulting in limited or inhibited pathogen spread, programmed cell death, or hypersensitive response (HR), often followed by systemic signalling and systemic acquired resistance (SAR) [25]. In susceptible hosts, basal defences are initiated but are not fast or effective enough to limit pathogen growth, allowing the pathogen to replicate and spread systemically. Activated defence responses result from several possible signalling pathways, including reactive oxygen species (ROS), signalling molecules, and pathogenesis-related proteins (PR proteins), which lead to biochemical and morphological alterations in the host plant such as cell-wall reinforcement and transmembrane reconfiguration [26, 27]. The outcome between susceptibility and resistance depends on the pathogen-host genotype combination [28], speed of host response, and specific virus pathogenicity determinants which recognize and interact with host-specific proteins [23, 29]. As mentioned previously, with plant viruses, including geminiviruses, the pathogen has to suppress basal immune systems such as RNA silencing. Many virus-encoded proteins act as host defence response suppressors such as HC-PRO of potyviruses and AC2, AC3 and AC4-ORF-encoded proteins of geminiviruses [30–32].

Following virus infection, transcriptional reprogramming takes place at a global level, both temporally and spatially within the plant leaves and other organs, and depending on the collective outcome, a resistance or susceptible response is initiated [19, 33–35]. Disease is usually manifested due to virus-induced physiological changes and direct interaction between virus and host proteins. Once a virus has successfully entered and completed replication in initial cells, it spreads via plasmodesmata through the leaf tissue or other tissues, and colonizes distal tissues in the plant, leading to a susceptible interaction, with disease as the final outcome [36, 37]. Geminivirus proteins have been shown to interact with a diverse set of host factors in *Arabidopsis thaliana*, *Solanum lycopersicum* and *Nicotiana benthamiana* [18, 38, 39] (reviewed in Jeske, 2009) [40]. Geminiviruses have been implicated in many host-responsive processes such as transcriptional regulation, DNA replication, control of the cell cycle, cell proliferation and differentiation, and macromolecular trafficking in whole plants [31, 41, 42]. In addition, the geminivirus AC2, AC3 or AC4 –encoded proteins have been implicated as a pathogenicity factor that assists in infection [24, 31, 32] and AC3 has been shown to affect transcriptional activation of a NAC transcription factor [32]. In particular, the geminivirus, *Tomato yellow leaf curl virus* (TYLCV) has been shown to interact with a NAC domain protein in a yeast two-hybrid system, where overexpression of the NAC transcription factor causes enhanced viral replication [43].

Gene expression technologies, such as microarrays represent a well-established technology and have been widely exploited in the last years leading to a vast amount of gene expression information, particularly in the area of host-pathogen interactions [33, 44–46]. To date, only two comprehensive full-genome microarray studies have been performed in *Arabidopsis* with geminiviruses, namely *Cabbage leaf curl virus* (CaLCuV) at 12 dpi [31], and more recently SACMV at 14, 24 and 36 dpi [47]. More recently, a third global microarray study was conducted in tomato using Agilent Tomato Gene Expression Microarrays, where the transcriptional changes induced by the phloem-limited geminivirus *Tomato yellow leaf curl Sardinia virus* (TYLCSV) was investigated [48]. In another geminivirus study by Eybishtz et al. [49], a reverse genetics approach was applied to identify genes involved in *Tomato yellow leaf curl virus* (TYLCV) resistance. Approximately 70 different cDNAs, representing genes preferentially expressed in a resistant (R) tomato line compared to a susceptible line from the same breeding program, were identified. Furthermore, a hexose transporter gene LeHT1 was shown to be up-regulated upon infection in R plants and its silencing in R plants led to the collapse of resistance [50]. In another recent study, the transcriptome reprogramming in leaves of susceptible (S) and R plants at 0 and 7 dpi after TYLCV inoculation, using a 60-mer oligonucleotide microarray was investigated [51]. Upon TYLCV infection, the genes differentially expressed in So versus Ro plants (before infection) were also those differentially expressed in Si vs Ri (after infection) plants. In Ro plants, the highly expressed genes were related to biotic stress, jasmonic acid and ethylene biosynthesis, signal transduction, and RNA regulation and processing. Furthermore, upon infection of R plants (Ro versus Ri), the number of differentially expressed genes was reported to be three times higher compared to the number of differentially expressed genes upon infection of S tomatoes (So versus Si) pointing to a strong response of R plants to the virus, which may be related to the resistance phenotype.

In recent years, the introduction of next-generation sequencing (NGS) has provided new and innovative ways to speed up the identification of large numbers of genes in many plant and animal species, particularly those under biotic and abiotic stresses [13, 15, 52, 53]. NGS has become the new method of choice for gene expression experiments as it is an extremely sensitive technique which has allowed for global analyses of exceptionally large datasets from transcriptomic, proteomic, metabolic, regulatory and developmental pathways to create networks that categorize interactions and function of organs or molecules at varying complexity levels [52]. Several NGS platforms have emerged, including Roche 454, Illumina GA, and ABI SOLiD [54–57]. GS-454 sequencing for example was used recently to analyse the transcriptome of symptomatic and recovered leaves of pepper infected with the geminivirus PepGMV [15].

Several recent studies have been reported in cassava using genomic tools. EST and cDNA libraries have been constructed in cassava for identification of abiotic/biotic responsive genes [58–62] or to analyse gene expression in response to the bacterial pathogen *Xanthomonas axonopodis* [63]. For example, a transcriptome analysis using an oligomicorarray representing ±20,000 cassava genes revealed 1300 abiotic drought stress related genes up-regulated in cassava [64]. A draft cassava genome is now publically available through phytozome (http://www.phytozome.net/cassava) [65]. Moreover, the function of homologous genes in *Arabidopsis* (http://www.arabidopsis.org/) can be used to predict the function of cassava genes. Cassava belongs to the family *Euphorbiaceae*, and its genome comprises an estimated 770 Mb [66]. A draft genome assembly and partial annotation of cassava from a single accession AM560-2 was released at the end of 2009 [65]. The genome assembly is in 12, 977 scaffolds, with a total scaffold length of 532.5 Mb. Ninety six percent of the putative transcripts from the publically available cassava EST database (http://cassava.igs.umaryland.edu/cgi-bin/index.cgi) can be mapped to the genome, making this a powerful tool for functional genomic studies. To date 30,666 protein-coding loci have been predicted, and the cassava genome can easily be aligned to soybean, castor bean, *Arabidopsis*, and rice. In addition to the cassava draft genome, there are also a number of additional cassava EST resources available through different databases (reviewed in [67]). Some of these include the availability of more than 80 000’s ETS through Genbank as well as two further large EST libraries containing between 20 000 and 30 000 Sanger reads which was generated as a collaborative effort between RIKEN (Rikagaku Kenkyusho—Institute of Physical and Chemical Research, Japan), and CIAT (Centro Internacional de Agricultura Tropical).

Most recently, a NGS (Illumina Solexa) gene profiling study was performed on cassava infected with *African cassava mosaic virus* (ACMV), and 3,210 differentially expressed genes were identified, with the study focusing on photosynthesis-related gene expression [68]. Despite this report, comprehensive genome-wide expression profiling data for cassava in response to viral pathogens remains lacking, and this research provides for the first time a full comparative analysis of global geminivirus-responsive transcriptomes in a susceptible and tolerant landrace, at three time points post infection. Applying all the available genetic resources recently made available, the aim of this study was to elucidate the gene expression responses of susceptible (T200) and tolerant (TME3) cassava landraces to SACMV infection at three stages during the course of infection, namely, pre-symptom (12 dpi), symptomatic (32 dpi) and late infection (67 dpi). These landraces were selected as T200 is a highly susceptible commercially grown South African landrace that is high in starch (unpublished), while TME3 is an established landrace in West Africa, known to be tolerant to cassava begomoviruses [9]. SOLiD (Applied Biosystems) transcriptome profiling data from six cDNA libraries derived from SACMV-infected apical leaf tissue, and six from *Agrobacterium* mock-inoculated controls was successfully generated. RNA-Seq data generated from the SOLiD platform was assembled and reference-based mapping against the cassava genome was performed. In total, 4181 and 1008 differentially expressed genes (DEGs) were identified in T200 and TME3, respectively, across all 3 time points, and their biological functions were established through gene ontology (GO) annotation and Kegg pathway analysis. Real-time qPCR was used to validate RNA-seq data and genes of interest selected for further analysis. Comparisons of expression patterns between T200 and TME3 at three time points post inoculation (12, 32 and 67 dpi), compared to mock inoculated tissue, demonstrated that differential responses to SACMV infection occur between the susceptible T200 and tolerant TME3 cultivars, and also between time points. TME3 had a significantly lower number of altered transcripts compared with T200. Comparisons were made to a previous study, conducted by Pierce and Rey, 2013 [47], in the susceptible *Arabidopsis*-SACMV pathosystem, and results uncovered similar and different global patterns or trends in differentially expressed genes between the two hosts.

## Results and discussion

### SACMV infectivity assays in T200 and TME3

**Figure 1:**
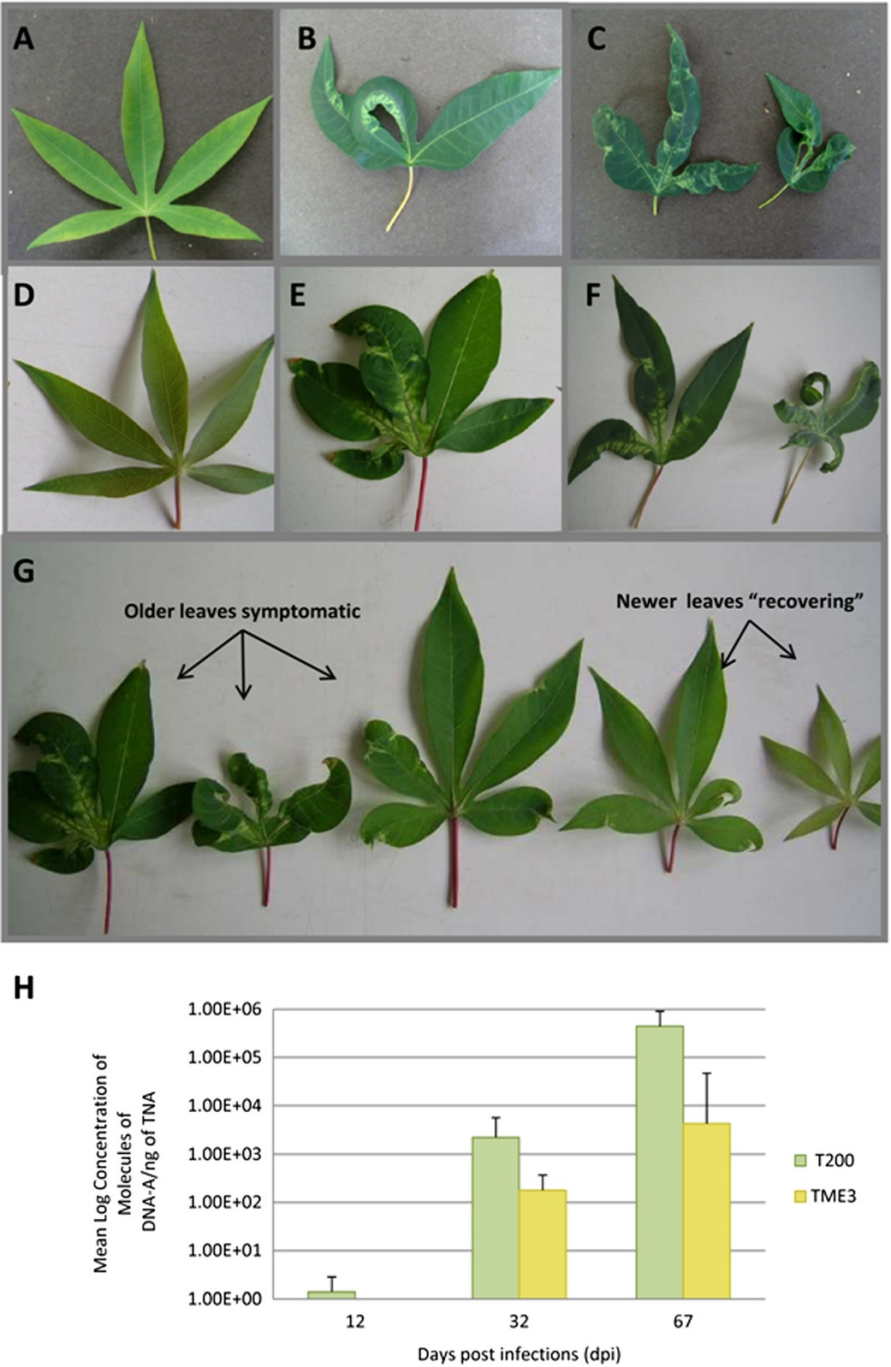
**T200 and TME3 infected leave tissue was evaluated for the development of symptoms over a 67 day period.** Leaves for both T200 **(A)** and TME3 **(D)** at 12 dpi (early infection) appear symptomless. Leaves at 32 dpi for T200 **(B)** and TME3 **(E)** display characteristic mosaic, distortion of leaf margins and leaf curling. Leaves at T200 **(C)** and TME3 **(F)** are severely reduced in size. Newer emerged leaves on some cassava TME3 **(G)** plants (on average, 2 out of every 6), displayed an attenuation in symptoms and almost appear symptomless. Viral titre from SACMV-–infected T200 and TME3 leaf tissue was measure using qPCR and is reported as the mean Log concentration of DNA-A molecules/ng TNA for 12, 32 and 67 dpi in infected leaf tissue samples **(H)**.

Following agro-inoculation of T200 and TME3, plantlets were monitored over a 67 day period for symptom development (Figures 1A-G) and concentration of virus (Figure 1H). Based on trial infections, time points chosen for this study represent different stages of infection where 12 dpi represents early infection (pre-symptomatic), 32 dpi represents active systemic virus replication and movement (symptomatic) and 67 dpi represents a later infection stage (persistently symptomatic in T200 and recovery in TME3). The symptom severity score index (1–5) [69] was used as a guideline for the assessment of symptom development in cassava plants. The mean (n = 6) symptom severity scores calculated for T200 at 12, 32 and 67 dpi showed increasing development of symptoms over time. At 12 dpi, the mean symptom severity score indicated that plants were asymptomatic (score of 1) (Figure 1A). By 32 dpi, symptoms developed uniformly in SACMV-infected plants which displayed typical mosaic and mild leaf distortion (Figure 1B) associated with CMD infection and the mean symptom severity score of 3.5 indicated that plants were showing moderate symptoms. At 67 dpi, plants were fully symptomatic with severe leaf symptoms (Figure 1C) and the mean symptom severity score of 4. West African landrace TME3, possessing a marker-linked CMD2 resistance gene [10], is reported to exhibit resistance to CMD. From our infectivity assay results, we observed that TME3 does not show early “resistance” but rather becomes infected by SACMV (using agroinoculation with SACMV infectious clones), and symptoms appear later (~1 week) compared with T200, with plants exhibiting a recovery phenotype after approximately 55–70 days onwards, resulting in new leaves displaying asymptomatic or mild symptoms. Symptom phenotype was confirmed by both severity indexing of infected TME3 in addition to viral titres (described later). The mean (n = 6) symptom severity scores were calculated for TME3 at 12, 32 and 67 dpi, and leaves were shown to be asymptomatic at 12 dpi up to ~21 dpi (Figure 1D). TME3 showed a different trend to that observed in T200 plants, where leaf symptoms, while visible at 32 dpi (Figure 1E), peaked later than 32 dpi, showing mosaic and distortion of leaf margins from 32–55 dpi (score 3–3.5) (Figure 1E-F). At 67 dpi (Figure 1G), TME3 plants were displaying slightly milder symptoms as compared to T200 at the same time point. Newly emerging leaves on plants showed either an attenuation of symptoms and had lower symptom severity scores (between 0 and 1) at 67 dpi (Figure 1G), or displayed no symptoms.

### Real –time qPCR measurement of SACMV viral titres in T200 and TME3

The concentrations of SACMV DNA-A were measured in infected and mock-inoculated T200 and TME3 plants at 12, 32 and 67 dpi (n = 6) (Figure 1H). A technical replicate was included for each biological replicate. For susceptible T200, the concentrations of DNA-A at 12 dpi were extremely low and almost undetectable (0.14 × 10^1^ SACMV molecules/ng total nucleic acid (TNA)), while at 32 and 67 dpi, 2.19 × 10^3^ and 4.43 × 10^5^ SACMV molecules of DNA-A/ng TNA were detected. In comparison, for tolerant cultivar TME3, viral loads of DNA-A were significantly lower (p < 0.05) than those detected in T200 where no virus was detected at 12 dpi, and 1.79 × 10^2^ and 3.23 × 10^4^ SACMV molecules of DNA-A/ng TNA were present at 32 and 67 dpi, respectively (Figure 1H). Overall, viral load in T200 between 32 and 67 dpi was 10-fold higher than that observed in TME3 at the same time points. These concentrations correlated well with the mean symptom severity score recorded for both cultivars. The increase in virus titre in T200 over time may correlate with host gene suppression. A study by Pierce and Rey (2013) [47] using an *Arabidopsis*-SACMV pathosystem also demonstrated similar trends in virus load over time, but in cassava, SACMV replication levels were higher compared with *Arabidopsis* [47]. The higher SACMV replication levels observed in cassava T200 could be attributed to the fact that T200 is a natural host to SACMV, providing a more favourable replication-competent environment.

### SOLiD Transcriptome data for analysis of SACMV-infected cassava

Sequence reads were obtained using the SOLiD v4 sequencing platform in order to generate a gene expression profile of T200 and TME3 infected with SACMV. The sequencer was run in the paired end mode with 50 bp forward (F3) and 35 bp reverse (F5) tags. Forward and reverse pairs were mapped to reference genome *Manihot esculenta* 147 available through phytozome (http://www.phytozome.net/cassava) and percentages were calculated for each F3 and F5 mapping combination for T200 and TME3 libraries (Additional file 1). The BAM files generated for the T200 and TME3 libraries are all publically available through the Sequence Read Achive (SRA, (http://www.ncbi.nlm.nih.gov/sra) using the BioProject accession number: PRJNA255198 [70].

In general, for the TME3 tolerant library, an average of 23.41% of both the forward and reverse reads mapped to the reference sequence, 22.74% of the forward F3 reads mapped, but only 6.50% of the reverse F5 read mapped. Furthermore, 47.19% of F3 + F5 reads did not map at all. Similarly, for T200, an average of 23.79% of both the forward and reverse reads mapped to the reference sequence, 22.19% of the forward F3 reads mapped but only 5.91% of the reverse read mapped. For T200, 48.11% of F3 + F5 reads did not map at all. The difference in F3 versus F5 mapping results from the actual SOLiD sequencing protocol which leads to a much higher percentage of F3 mapped reads compared to F5. Because the F5 reads are of lower quality, the aligner (Lifescope) preferentially uses the F3 quality scores in mapping to the reference genome. The fraction of unmapped reads may be due to the incompleteness of current cassava genome assembly in which thousands of scaffolds are still not interconnected, and also the lower quality of the F5 reads, as mentioned previously. Despite incomplete assembly of the cassava genome, the current status of gene annotation for the assembled scaffolds on phytozome is reliable, which makes this assembly useful for RNA sequencing alignment and analysis.

Normalization was carried out as an averaging geometric mean of replicates for each library. Normalized data was then imported into DESeq R software package where the counts for differentially expressed genes were calculated using the negative binomial distribution estimated from the complete dataset. Cassava transcripts identified as differentially expressed were annotated using the “M.esculenta_147_annotation_info” file available from phytozome and blasting against the *Arabidopsis* database (Additional file 2).

### Global gene expression profiling of T200 and TME3 in response to SACMV infection

**Figure 2:**
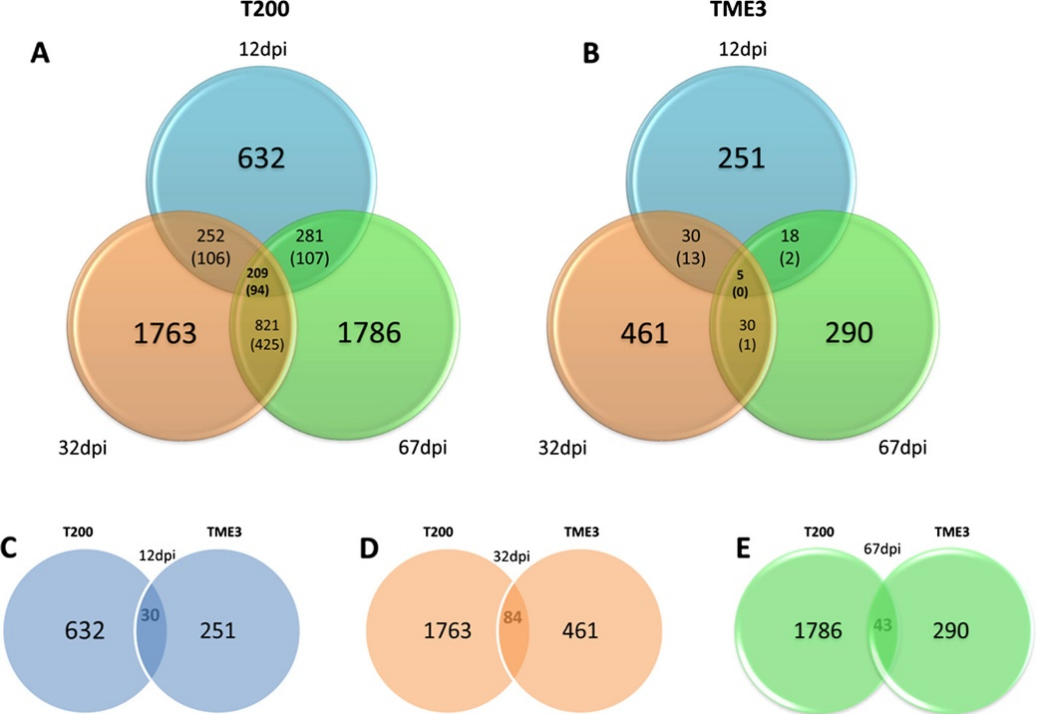
**Venn diagrams showing the differential distribution of up-regulated (>2.0-fold) and down-regulated (<2.0-fold) transcripts in SACMV-infected T200 (A) and TME3 (B) leaf tissues at three different time points post infection.** Comparisons of differentially-expressed transcripts between T200 and TME3 at 12dpi **(C)**, 32 dpi **(D)** and 67 dpi **(E)**. The values in the brackets indicate the number of genes downregulated between timepoints.

In order to quantify the differential expression of genes at 12, 32 and 67 dpi in susceptible T200 and tolerant TME3 landraces, the tag count for all genes at 12, 32 and 67 dpi versus the tag counts at the same time points in mock-inoculated samples were computed. This allowed the change in expression between SACMV-infected and mock-inoculated leaf tissue samples to be calculated at all three time points for both landraces. After statistical filtering of the data (log_2_-fold cut-off, p < 0.05), the total number of differentially expressed genes (DEGs) were identified as SACMV- responsive genes for T200 (Additional files 3, 4 and 5) and TME3 (Additional files 6, 7 and 8). These are depicted in the Venn diagram (Figure 2). Overall, the number of differentially expressed genes (DEGs) in tolerant TME3 infected with SACMV was significantly lower, over the 67 dpi period, than that observed for susceptible T200 plants. In T200, 632 DEGs were detected in apical leaves at early infection (12 dpi), where 417 genes were up regulated and 215 genes were down regulated (Additional file 3). At 32 dpi, this number increased to 1763 where 742 genes were up regulated and 1021 genes were down regulated (Additional file 4) and at 67 dpi, a total of 1786 DEGs were detected where 991 genes were up regulated and 795 were down regulated (Additional file 5). In comparison, for early response at 12 dpi, only 251 DEGs were detected in TME3 apical leaf tissue, where 63 were up regulated and 188 were down regulated (Additional file 6). At 32 dpi, 461 DEGs occurred where 294 genes were elevated and 167 were suppressed (Additional file 7), and at 67 dpi, 290 genes were altered where 88 genes were up regulated and 202 genes were down regulated (Additional file 8). In general, a shift from up-regulated genes at an early time point (12 dpi), to down-regulated genes in fully symptomatic leaves at 32 dpi is not uncommon in susceptible hosts, as large amounts of virus nucleic acid and proteins produced during cellular infection cause normal cellular processes to be redirected toward viral replication [35]. It was also evident that SACMV was able to maintain a high level of transcript repression as virus infection persisted (67 dpi), and because cassava is a vegetatively propagated crop, systemic infection can persist for months until harvest. Viruses have been shown to cause host gene shut-off in an attempt to inhibit broad spectrum defence responses activated by the plant [20, 37]. Although host shut-off was previously described as transient, more recently, Conti et al. [71] demonstrated that gene-specific and persistent shut-off was evident in *Nicotiana tabacum* upon *Tobacco mosaic virus* (TMV) infection, and similarly, in the *Arabidopsis*-SACMV study [47], persistent down-regulation of many genes across 3 time points post-infection was observed.

A comparison of consistently expressed transcripts across the three time points, and between each two time points was evaluated for T200 (Additional file 9) and TME3 (Additional file 10). For T200, 209 genes were consistently altered across the three time points (Figure 2A), while in comparison, only 5 were noted in TME3 (Figure 2B). In T200, 252 genes were common between 12 and 32 dpi, 281 genes were common between 12 and 67 dpi and 812 genes were common between 32 and 67 dpi (Additional file 9; Figure 2A). For TME3, the overlap was considerably smaller, where only 30 genes were common between 12 and 32 dpi, 18 genes between 12 and 67 dpi, and 30 genes between 32 and 67 dpi (Additional file 10, Figure 2B). Not withstanding the different genetic backgrounds between T200 and TME3, it was interesting to observe that very few shared genes, out of the total number altered by SACMV in the susceptible T200 and tolerant TME3 landraces, were observed. At 12 dpi only 30 genes were shared between T200 and TME3 (Figure 2C), while 84 and 43 were shared at 32 and 67 dpi, respectively. In T200, large numbers of transcripts involved in basal defence were down regulated, especially at 32 dpi (full systemic infection), which resulted in persistent virus infection and susceptibility. Some similar and different patterns in defence-related gene expression between T200 and SACMV-infected *Arabidopsis* [47] were noted, but in the tolerant phenotype TME3, suppression of 188 (74% of total altered) transcripts compared to T200 (34% of total altered transcripts) appeared at an earlier time point, 12 dpi, which suggests a more rapid response to SACMV. Also most notably at 67 dpi, 70% of transcripts were suppressed in TME3, which correlated to symptom recovery and drop in virus load (Figure 1).

### Gene Ontology clustering of SACMV-responsive genes in susceptible T200 and tolerant TME3 at 12, 32 and 67 dpi, and comparison with Arabidopsis

**Figure 3:**
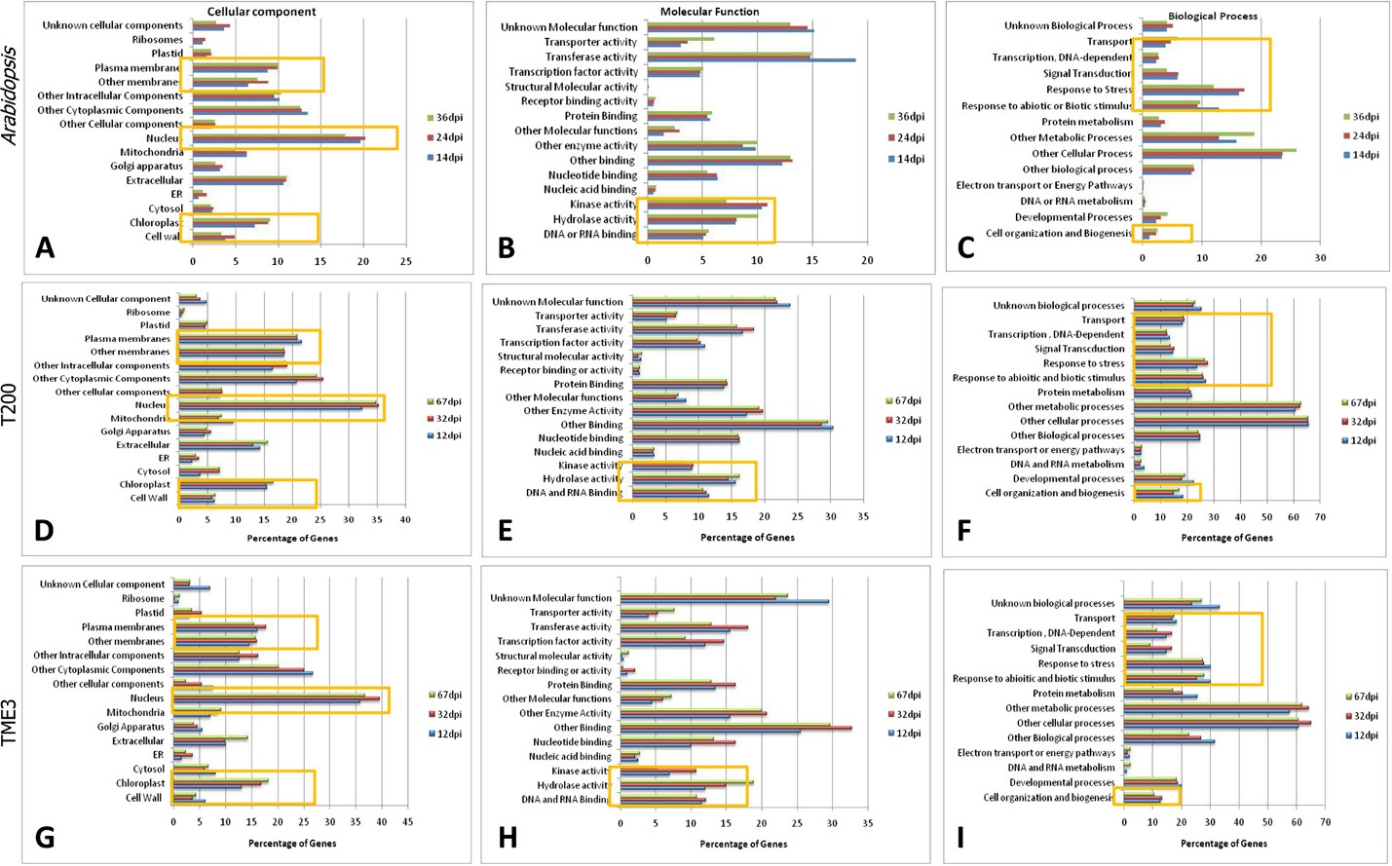
**GOSlim Functional characterisation of T200 and TME3 DEGs at 12, 32 and 67 dpi for cellular component (A,D,G), biological process (C,F,I) and molecular function (B,E,H).** Orange demarcated areas indicate the most significant changes in the percentage of DEG categories in Arabidopsis **(A,B,C)**, T200 **(D,E,F)** and TME3 **(G,H,I)**.

The Arabidopsis AGIs for the annotation of cassava transcripts were used to identify the functional enrichment of differentially expressed genes using Gene Ontology (GO) vocabulary available on TAIR 10 (http://www.arabidopsis.org/tools/bulk/go/index.jsp), at each time point (12, 32 and 67 dpi) for each cultivar. Transcripts were sorted into GoSlim term categories for molecular function, biological processes, and cellular component, and comparisons with a microarray expression study performed in SACMV-infected *Arabidopsis* (at 14, 24 and 36 dpi) [47] was undertaken (Figure 3A-I). Regardless of the host (cassava or *Arabidopsis*) and platform (NGS or microarray), both pathosystems displayed similar trends in differential gene function categories representing the highest number of transcripts (Figure 3). While infection progress in the annual host *Arabidopsis* was expectedly faster compared with the perennial host, cassava, comparisons between equivalent early, middle and late stages revealed a similar pattern for the two most over-represented categories in cellular component, namely nucleus (19.6%, 14.9%, 17.1%) and cytoplasmic component (13.4%, 11.9%, 15.7%) for *Arabidopsis* (Figure 3A), T200 (Figure 3D), and TME3 (Figure 3G), respectively. Interestingly, the plasmamembrane component was also highly represented in all three plant hosts (8.7%, 11.4% and 9.9% for *Arabidopsis*, T200, TME3, respectively). For biological processes, cell organization and biogenesis, responses to stress and biotic/abiotic stimuli, and other metabolic and cellular processes were all highly represented categories (*Arabidopsis*, T200, TME3; Figure 3C, F, I, respectively), as well noticeable changes in the chloroplast fraction in all three hosts. Transferase and kinase, and other enzyme activity demonstrated the most noticeable transcript changes for molecular function (*Arabidopsis*, T200, TME3; Figure 3B, E, H, respectively).

### Independent validation of SOLiD NGS results by real-time-qPCR

**Figure 4:**
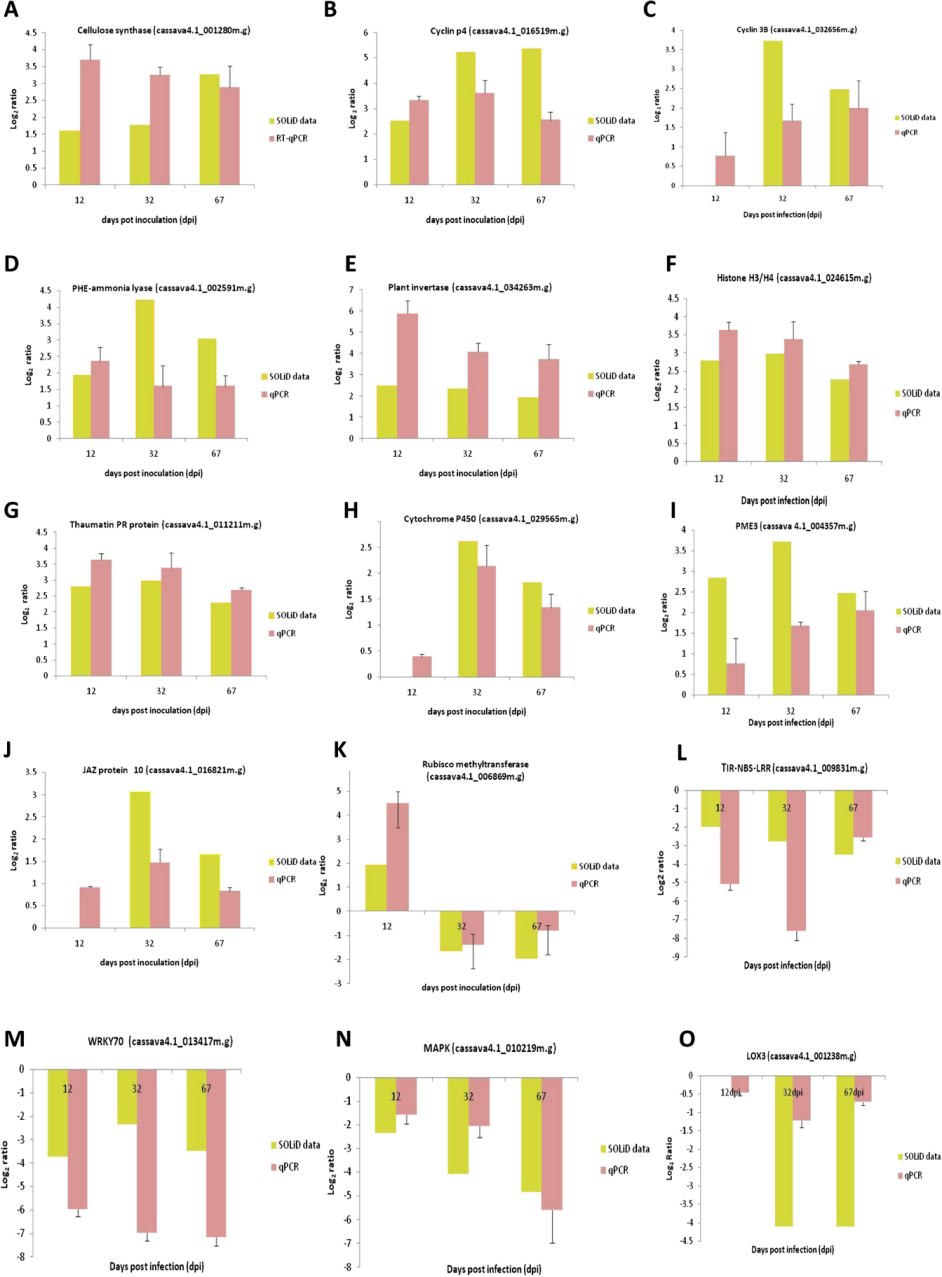
**RT-qPCR vs SOLiD Log**_**2**_ **gene expression ratios of fifteen genes (A-O) measured from SACMV leaf tissue at 12, 32 and 67 dpi in T200 and TME3.** Twelve genes were chosen for T200 **(A-L)** and 3 for TME3 **(M-O)**. The expression of each gene was normalized to endogenous *UBQ10*.

To validate the SOLiD RNA-seq data, RT-qPCR was performed on fifteen (12 from T200 and 3 from TME3) genes that were significantly changed upon SACMV infection (>2-fold, p < 0.05). The expression levels for cellulose synthase, cyclin p4, PHE-ammonia lyase, plant invertase, thaumatin PR protein, cytochrome P450, JAZ protein 10, Rubisco methyltransferase, WRKY70, MAPK3, cyclin 3B, histone H3/H4, pectin methylesterase (PME3), lipoxygenase (LOX3) and TIR-NBS-LRR (Figures 4A-O) were independently validated on cDNA samples (at 12, 32 and 67 dpi) from the SOLiD RNA-seq study. The standard curve method [72] was used to determine expression values for each target gene from SACMV- infected leaf tissue at each time point in relation to the expression of the same target in mock-inoculated leaf tissue. Relative expression values for each target gene were then expressed as a Log_2_ ratio of target gene expression level to *UBQ10* expression level measured in the same cDNA sample. Therefore, expression levels are presented as the relative Log_2_ ratio of the infected cassava leaf tissue sample compared with the control mock-inoculated sample at each time point. Results showed that computational predictions of differential expression were validated. Although, in general, RT-qPCR was expectedly more sensitive, all fifteen genes showed correlated Log_2_ gene expression patterns (up or down regulated), in agreement with those observed in SOLiD sequencing data.

### Differentially expressed gene patterns in T200 and TME3 in response to SACMV infection

Notwithstanding the economic importance of cassava, particularly in developing countries, it has received little attention in the scientific community in contrast to the model species *Arabidopsis thaliana* and *Nicotiana benthamiana*, or crops such as rice, potato and tomato. There are only a handful of biotic stress-response global gene expression studies that have been carried out in cassava [60, 63, 68] and most recently, an abiotic study demonstrating the effect of cold stress on the apical shoots of cassava was reported [73]. A gene expression profile of *Xanthamonas* infection in cassava has also been reported [63], and more recently a Roche 454 GS20 platform was applied to uncover transcriptome differences in recovered and symptomatic leaves of geminivirus-infected pepper [15]. To date, only one other NGS full transcriptome study has been carried out in cassava infected with a geminvirus [68]. Liu et al. [68] made use of the Illumina platform in order to dissect transcriptional changes in photosynthesis that occur in cassava leaves infected with ACMV. Here, we present comparative transcriptome data between a susceptible and tolerant cassava landrace in response to a geminivirus, SACMV, at three time points post infection. Cassava is a vegetatively propagated perennial crop, and virus persistence occurs throughout the life-cycle of the plant until it is harvested, therefore in cassava one anticipates a continuous fluctuation in host responsive genes as the virus spreads systemically to new apical leaves, where geminiviruses prefer to replicate [39, 40]. Therefore, there would be dynamic changes in activation and suppression of responses during the virus-host interaction where the host attempts to mount a basal defence and the geminivirus overcomes this by suppression. In order to avoid inconsistencies across older leaves and to minimize spatial variations, transcriptome changes were consistently monitored in upper leaves below the apex, where SACMV is actively replicating. While there were expected differences in the transcriptomes between uninfected T200 and TME3, the data in this study clearly demonstrates transcriptional activation or repression of a large number of SACMV-responsive genes in both susceptible and tolerant landraces (Additional files 3, 4, 5, 6, 7, 8, 9 and 10). These patterns of expression are particularly interesting as, notwithstanding some shared similarities, they differ between susceptible T200 and tolerant TME3 landraces. However what clearly emerges is that, in addition to virus-specific responses, many general biotic stress responses in cassava to a DNA virus are similar to other susceptible hosts and RNA viruses [37–39, 44].

Due to the large wealth of data generated in this study, we targeted genes that were common in both landraces but showed differing expression patterns at various time points post infection, or common/unique genes in GO categories that were over- or under-represented, and that have been shown to play a role in plant virus-host interactions. Some of these groups include metabolic pathways, defence responses, transcription factors, R genes, histone/DNA methylation-associated genes, and cell-wall and plasmadesmata associated genes. For the selected differentially DEGs discussed below, we scrutinized the uninfected (mock-inoculated) T200 and TME3 data (Additional file 11) to ascertain differences in transcript quantifications between the susceptible and tolerant landraces. Not surprisingly, we found that there were differences in the transcript frequency between T200 and TME3 for a number of genes involved in resistance, defence, photohormone signalling and those associated with the cell wall and plasmadesmata. We predicted that the number of R genes to be higher in tolerant TME3 than T200, however, we observed that the transcript frequency for a majority of the genes were lower (Additional file 11). For genes associated with defence, particularly many heat shock proteins, we observed that the transcript numbers in TME3 was higher compared to T200 (highlighted in yellow, Additional file 11). These differences observed could indicate that these two transcriptomes are already predispositioned or ‘primed’ to respond differently to virus infection.

Many common genes were differentially expressed over all 3 time points post-infection during the SACMV course of infection progression in T200 (Additional file 9). Induced transcripts such as pectin lyase superfamily proteins and plant invertase/pectin methylesterase inhibitor superfamily proteins, involved in cell wall degradation were induced in T200, and may play a role in long distance movement and exit from the phloem [18, 44]. Additionally, transcripts involved in secondary metabolism such as serine carboxypeptidase-like 45 and those involved in protein/peptide degradation such as eukaryotic aspartyl protease family proteins which are involved in protein/peptide degradation were also up-regulated across time points. Transport genes showing differential expression were those genes involved in cation transport such as the up-regulated potassium transporter 2 protein, whereas the heavy metal transport/detoxification superfamily protein was down-regulated across the 3 time points. Sugar transport proteins such as the major facilitator superfamily protein were up-regulated, whereas Cytochrome P450, family 71, subfamily B, polypeptide 37 and Cytochrome P450, family 76, subfamily G, polypeptide 1, all involved in electron transport, were down-regulated across all three time points. A very interesting finding was the up-regulated cyclin P4:1 gene in T200, which is involved in the cell cycle and DNA processing, and geminiviruses have been shown to interfere with cell cycling in a host [31]; discussed in detail in Pierce and Rey (47).

### KEGG pathway analysis of SACMV-responsive genes

Virus infection has been shown to disrupt the highly ordered primary metabolism of the host plant. KEGG pathway analysis was carried out for T200 and TME3 for commonly regulated transcripts using DAVID (http://david.abcc.ncifcrf.gov/). Details of metabolites and p-values are depicted in Table 1 and Additional file 12. Noticeably, neither T200 nor TME3 exhibited any changes in transcripts associated with metabolic pathways early after infection (12 dpi), except for flavanoid biosynthesis in T200 (Table 1). TME3 displayed a small set of genes (7.9%) across time points that mapped to several pathways, notably stilbenoid, diarylheptanoid and gingerol biosynthesis, pentose and glucuronate interconversions and starch and sucrose metabolism (Table 1). On the other hand, T200 collectively had 11% of differentially expressed transcripts mapping to flavanoid biosynthesis (10 genes, P = 1.2E-9), biosynthesis of phenylpropanoids (18 genes, P = 0.01), phenylpropanoid biosynthesis (9 genes, P = 0.014), and stilbenoid, diaryheptanoid and gingerol biosynthesis (6 genes, P = 0.051) (Additional file 12). Common up-regulated gene transcripts in cassava T200 across 3 time points, involved primarily in metabolism, were EMB3004, MEE32 (dehydroquinate dehydratase/ shikimate dehydrogenase) and UGT84A1 which are involved in C-compound and carbohydrate metabolism. In addition, genes such as EMB3004, MEE32 and CYP75B1, D501, TT7, involved in secondary metabolism, were induced across time points, and haloacid dehalogenase (HAD) and PERK10 (Proline-rich Extensin-like Receptor Kinase 10), that are involved in phosphate metabolism, were down-regulated across time points. HAD is also involved in metabolism of energy reserves such as glycogen and trehalose. In comparison, *Arabidopsis* showed a similar pattern of low numbers mapping to metabolic pathways at 14 dpi, while at 24 and 36 dpi, 5.6% and 7.1% of altered genes mapped to metabolic pathways (Table 1).

**Table 1:** **Kegg pathway analyses of differentially expressed metabolites in SACMV-infected Arabidopsis, and cassava T200 (susceptible) and TME3 (tolerant)**

Metabolite pathway	% genes mapping in *Arabidopsis*			% genes mapping in cassava T200			% genes mapping in cassava TME3		
	14 dpi	24 dpi	36 dpi	12 dpi	32 dpi	67 dpi	12 dpi	32 dpi	67 dpi
Tropane, piperidine and pyridine alkaloid biosynthesis	0.7		0.4						
Phenylpropanoid biosynthesis	1.2	1.3	1.6		1.1	1.1			
Phenylalanine metabolism	1.0	1.0	1.1						
Nitrogen metabolism	0.7		0.6		0.6	0.6			
Methane metabolism		1.0	0.8						
Glycerolipid metabolism			0.4						
Flavanoid biosynthesis				0.7	0.7	0.7			
Stilbenoid, diarylheptanoid and gingerol biosynthesis					0.7	0.7		1.5	
Pentose and glucuronate interconversions									1.10
Starch and sucrose metabolism					0.8	0.8			2.6
Pantothenate and CoA biosynthesis					0.3	0.3			
Biosynthesis of plant hormones								3.2	
alpha-Linolenic acid metabolism								2.0	
Limonene and pinene degradation								1.2	
Arabidopsis	14 dpi (26 genes of 4067 map to pathways) (0.63%)								
	24 dpi (40 genes of 711 map to pathways) (5.60%)								
	36 dpi (71 genes of 996 map to pathways) (7.1%)								
Cassava T200 32 and 67 dpi	Alpha,alpha-trehalose-phosphate synthase [UDP-forming] 1 (AT1G78580)								
	Beta-galactosidase 13 (AT5G44640)								
	Beta-galactosidase 17 (AT2G44480)								
	Endoglucanase 16 (AT3G43860)								
	Glucose-1-phosphate adenylyltransferase large subunit 2, chloroplastic (AT1G2768)								
	Glucose-1-phosphate adenylyltransferase large subunit 2, chloroplastic (AT1G27680)								
	Glucose-1-phosphate adenylyltransferase (AT1G19920)								
	Pectinesterase 4 (AT2G47030)								
	Phosphorylase (AT3G29320)								
	UDP-glucuronate 4-epimerase 6 (AT3G23820)								
Cassava TME3 32 dpi	12-oxophytodienoate reductase 2 (AT1G76690)								
	12-oxophytodienoate reductase 3 (AT2G06050)								
	2-C-methyl-D-erythritol 4-phosphate cytidylyltransferase, chloroplastic (AT2G02500)								
	3-hydroxy-3-methylglutaryl-coenzyme A reductase 1 (AT1G76490)								
	4-coumarate--CoA ligase-like 5 (AT1G20510)								
	(Unknown AT1G17420)								
	Allene oxide synthase, chloroplastic (AT5G42650)								
	Jasmonate O-methyltransferase (AT1G19640)								
	Probable 1-deoxy-D-xylulose-5-phosphate synthase, chloroplastic (AT4G15560)								

One of the most interesting discoveries, which have not been extensively reported in cassava before, was the mapping of several flavanoid and phenylpropanoid genes involved in T200 infection, which were prominently altered at 32 dpi and maintained at 67 dpi. Genes mapping to these pathways included flavonol synthase (cassava4.1_011509m.g), UDP-glycosyltransferase (cassava4.1_005848m.g), chalcone synthase (cassava4.1_009206m.g, cassava4.1_009295m.g, cassava4.1_009402m.g) and phenylalanine ammonia lyase (cassava4.1_002591m.g, cassava4.1_002709m.g, cassava4.1_034377m.g). Furthermore, these genes were all found to be highly induced with expression ratios in the range of Log_2_ 1.95 – Log_2_ 4.45. Flavanoids and phenylpropanoids have been shown to play a role in early responses to pathogens [74, 75]. Phenylalanine ammonia lyase (PAL) is an enzyme that catalyzes the first and most important step in the phenylpropanoid pathway. Several lines of evidence indicate that PAL may participate in defending host plants against invading pathogens, and is often associated with the hypersensitive response (HR). This has been shown in a very early study conducted by Pallas *et al.* (1996) [20], where PAL-suppressed tobacco leaves did not result in the induction of downstream PR proteins in systemic leaves which therefore impaired an active defence response against TMV. More recently, Hoa *et al.* (2011) [76] demonstrated that PAL was highly induced (5.8-fold) in a resistant rice variety early hours after infection with *Rice stripe virus*, but not in a susceptible variety, suggesting that PAL plays a defence response. Similarly, the silencing of a pathogen-inducible UDP-glycosyltransferase in tobacco resulted in the depletion of UDP-glycosyltransferase in tobacco which enhanced oxidative stress and weakened resistance of silenced tobacco plants to TMV infection [77]. We, however, observed the activation of PAL, CHS and UDP-glycosyltransferase only at middle to late stages of infection in T200 (32 and 67 dpi), which is not unexpected as T200 is highly susceptible and unable to successfully mount an effective resistance response. The expression of PAL and CHS in particular was sustained across the time points, and it is not uncommon for a host to continue to mount basal immune responses throughout infection, albeit not timeously or sufficiently to effective limit replication and spread. In the SACMV-*Arabidopsis* study [47], PAL and peroxidase also continued to be highly expressed over early, middle and late stages of infection. In contrast in TME3, there appeared to be no basal defence response at 12 dpi related to secondary metabolites, and 74% of altered transcripts were down-regulated. It has been hypothesized from other studies, that plant hosts that suppress disease responses in a regulated manner, resulting in delayed or mild disease symptoms may be regarded as tolerant [78].

### Differential regulation of resistance (R) associated gene homologues in T200 and TME3

Transcript quantification showed that T200 had a far greater change in the number of differentially expressed genes as well as the magnitude of expression changes across time points compared with TME3 (Additional files 3, 4, 5, 6, 7, 8, 9 and 10). However one of the most noticeable observations made with regard to the transcript data, was the consistent down-regulation of several disease-associated resistant (R) gene homologues in SACMV-infected T200, and up-regulation in TME3 at later time points (Additional file 13). Seventy differentially expressed R gene homologues belonging to class I-IV [79] were identified in T200 and TME3. Notably, in TME3, few R gene homologues were altered, and all R genes were up-regulated at 32 (8 genes) and 67 (2 genes) dpi, corresponding to recovery. In contrast, in susceptible T200, 67 of the 70 identified R gene homologues were differentially expressed, with some overlaps at the three time points, but many uniquely altered at each dpi. Twenty two and forty eight R genes were down-regulated at 32 and 67 dpi, respectively, which correlates to high viral load and severe symptoms in T200 (Figure 1). Of these identified R gene homologue classes, 15 belonged to class I (Table 2), and interestingly only one class II (CC-LRR-NBS) (cassava4.1_014150m.g) R gene was identified and that was downregulated in T200 at 67 dpi. At early infection between 12 and 32 dpi only one TIR-NBS-LRR R gene was suppressed in T200. Two TIR-NBS-LRR class R genes were uniquely up-regulated in TME3 at 32 dpi, but were not detected in T200. A single TIR-NBS-LRR (R) gene (cassava4.1_009831m.g) was repressed across all three time points post-infection in T200, and several TIR-NBS-LRR (class I) R genes at 32 and 67 dpi (Table 2). Additionally, down-regulation of several NB-ARC domain-containing disease resistance proteins, leucine-rich receptor-like protein kinases and leucine-rich repeat transmembrane protein kinase family proteins, were observed in T200 (Additional file 13).

**Table 2:** **Selected differentially expressed (log**_**2**_ **-fold) genes in T200 and TME3 used for further discussion in this paper**

Gene	Cassava accession	Arabidopsis AGI accession	12 dpi		32 dpi		67 dpi	
			Log _2_ fold	p-value	Log _2_ fold	p-value	Log _2_ fold	p-value
**T200 - Class I resistance genes**								
Disease resistance protein (TIR-NBS-LRR class) family	cassava4.1_009831m.g	AT5G18350.1	−1.98336		−2.74964	4.62E-04	−3.16827	1.44E-04
Disease resistance protein (TIR-NBS-LRR class) family	cassava4.1_025981m.g	AT4G16960.1	−1.93152		-	-	-	-
Disease resistance protein (TIR-NBS-LRR class)	cassava4.1_006736m.g	AT1G69550.1	-	-	−3.93415	2.18E-06	−4.51391	2.55E-07
Disease resistance protein (TIR-NBS-LRR class) family	cassava4.1_000944m.g	AT4G12010.1	-	-	−2.40348	1.58E-03	−3.38156	3.74E-05
Disease resistance protein (TIR-NBS-LRR class) family	cassava4.1_000534m.g	AT5G36930.2	-	-	−2.04993	1.14E-02	−2.16756	1.24E-02
Disease resistance protein (TIR-NBS-LRR class), putative	cassava4.1_000331m.g	AT5G17680.1	-	-	−1.80402	1.48E-02	−2.20612	4.30E-03
Disease resistance protein (TIR-NBS-LRR class) family	cassava4.1_001210m.g	AT3G44480.1	-	-	−3.89602	2.04E-05	−3.88410	2.74E-05
Disease resistance protein (TIR-NBS-LRR class)	cassava4.1_007699m.g	AT1G69550.1	-	-	−3.68973	8.52E-06	−3.83451	5.23E-06
Disease resistance protein (TIR-NBS-LRR class), putative	cassava4.1_031642m.g	AT5G17680.1	-	-	−2.75917	8.32E-04	−3.88542	1.83E-05
Disease resistance protein (TIR-NBS-LRR class) family	cassava4.1_032672m.g	AT4G12010.1	-	-	−2.28131	3.04E-03	−3.47964	2.50E-05
Disease resistance protein (TIR-NBS-LRR class) family	cassava4.1_017691m.g	AT3G04220.1	-	-	−3.96304	1.78E-06	−4.09620	3.13E-06
**TME3 - Class I Resistance Genes**								
Disease resistance protein (TIR-NBS-LRR class), putative	cassava4.1_031334m.g	AT5G17680.1	-	-	1.93438	0.016708	-	-
Disease resistance protein (TIR-NBS-LRR class) family	cassava4.1_023684m.g	AT5G41750.2	-	-	2.59734	0.027843	-	-
**T200 - Histone-related genes**								
Histone H4	cassava4.1_029975m.g	AT2G28740.1	2.92352	0.03976	-	-	2.92352	2.66E-02
Histone H2A 8	cassava4.1_018866m.g	AT2G38810.2	2.28609	0.04821	-	-	-	-
Histone superfamily protein	cassava4.1_019888m.g	AT1G07820.1	1.74066	0.04304	1.74066	1.86E-02	-	-
Histone superfamily protein	cassava4.1_018611m.g	AT1G08880.1	-	-	3.68140	5.09E-03	-	-
Histone superfamily protein	cassava4.1_028744m.g	AT3G27360.1	-	-	2.48875	5.18E-03	-	-
Histone H2A 10	cassava4.1_026667m.g	AT1G51060.1	-	-	2.03029	9.65E-03	2.03029	8.78E-03
Histone superfamily protein	cassava4.1_030637m.g	AT3G27360.1	-	-	2.33165	1.04E-02	2.33165	4.00E-02
Histone superfamily protein	cassava4.1_024615m.g	AT3G27360.1	-	-	1.92352	4.00E-02	1.92352	1.77E-02
Histone H2A 7	cassava4.1_018569m.g	AT5G27670.1	-	-	-	-	1.68140	5.85E-03
Histone H2A 7	cassava4.1_018568m.g	AT5G27670.1	-	-	-	-	3.23092	4.02E-02
Histone H4	cassava4.1_019914m.g	AT2G28740.1	-	-	-	-	1.57560	1.63E-02
Histone superfamily protein	cassava4.1_018874m.g	AT3G27360.1	-	-	-	-	2.42885	2.81E-02
Histone H4	cassava4.1_019911m.g	AT2G28740.1	-	-	-	-	1.62150	4.14E-02
Histone H4	cassava4.1_019891m.g	AT2G28740.1	--	-	-	-	1.59130	4.43E-02
**TME3 - Histone-related genes**								
Histone acetyltransferase of the MYST family	cassava4.1_029570m.g	AT5G64610.1	-	-	-	-	−3.17558	0.03731
**T200 - WRKY genes**								
WRKY family transcription factor	cassava4.1_011089m.g	AT4G23810.1	−1.88970	0.03067	−1.88970	2.73E-04	−1.8897	8.23E-06
WRKY DNA-binding protein 70	cassava4.1_013417m.g	AT3G56400.1	−1.84402	0.04488	−1.8440	3.53E-03	−1.8440	1.22E-05
WRKY transcription factor family protein	cassava4.1_004372m.g	AT4G26640.2	2.34337	0.01006	-	-	-	-
WRKY DNA-binding protein 7	cassava4.1_010539m.g	AT4G24240.1	2.30203	0.02491	2.30207	8.07E-03	2.30207	5.26E-04
WRKY DNA-binding protein 40	cassava4.1_033249m.g	AT1G80840.1	-	-	−3.71714	7.65E-06	−3.7171	3.60E-03
WRKY family transcription factor	cassava4.1_011518m.g	AT4G11070.1	-	-	−2.5359	2.24E-05	−2.5359	7.70E-05
WRKY DNA-binding protein 33	cassava4.1_009059m.g	AT2G38470.1	-	-	−2.0461	2.43E-04	−2.0461	1.25E-03
WRKY family transcription factor	cassava4.1_007752m.g	AT2G38470.1	-	-	−2.6020	7.99E-04	−2.6020	2.87E-03
WRKY family transcription factor	cassava4.1_010768m.g	AT4G23810.1	-	-	−2.5635	8.93E-04	−2.5635	5.64E-05
WRKY DNA-binding protein 40	cassava4.1_011696m.g	AT1G80840.1	-		−2.6405	9.61E-04	-	-
WRKY DNA-binding protein 40	cassava4.1_024650m.g	AT1G80840.1	-	-	−2.47729	1.52E-03	−2.4772	6.29E-07
WRKY DNA-binding protein 57	cassava4.1_012575m.g	AT1G69310.2	-	-	−2.60063	2.15E-03	−2.6006	1.51E-02
WRKY DNA-binding protein 40	cassava4.1_012109m.g	AT1G80840.1	-	-	−2.24459	1.10E-02	-	-
WRKY DNA-binding protein 51	cassava4.1_016594m.g	AT5G64810.1	-	-	−3.8313	2.23E-02	-	
WRKY DNA-binding protein 70	cassava4.1_012154m.g	AT3G56400.1	-	-	−1.53590	2.26E-02	−1.5359	2.86E-03
WRKY DNA-binding protein 72	cassava4.1_004929m.g	AT5G15130.1	-	-	−3.22549	3.21E-02	-	-
WRKY DNA-binding protein 14	cassava4.1_030132m.g	AT1G30650.1	-	--	−4.0190	3.49E-02	-	
WRKY family transcription factor	cassava4.1_016397m.g	AT2G44745.1	-	-	2.21032	6.78E-03	-	-
WRKY DNA-binding protein 23	cassava4.1_011940m.g	AT2G47260.1	-	-	-	-	−2.9440	2.19E-02
WRKY family transcription factor	cassava4.1_024248m.g	AT4G01250.1	-	-	-	-	2.2109	4.08E-03
WRKY DNA-binding protein 35	cassava4.1_014297m.g	AT2G34830.1	-		-		2.78731	9.64E-03
**TME3 – WRKY Genes**								
WRKY DNA-binding protein 40	cassava4.1_011696m.g	AT1G80840.1	2.43773	4.31E-02	2.43773	0.00412	-	-
WRKY DNA-binding protein 40	cassava4.1_014368m.g	AT1G80840.1	4.24970	1.46E-02	-	-	-	-
WRKY DNA-binding protein 40	cassava4.1_024650m.g	AT1G80840.1	-	-	2.29778	0.00296	-	-
WRKY DNA-binding protein 40	cassava4.1_012109m.g	AT1G80840.1	-	-	2.08277	0.00524	2.08277	0.02056
WRKY family transcription factor	cassava4.1_004331m.g	AT1G62300.1	-	-	2.28487	0.01171	-	-
WRKY family transcription factor	cassava4.1_011518m.g	AT4G11070.1	-	-	1.89948	0.01235	-	-
WRKY DNA-binding protein 28	cassava4.1_011936m.g	AT4G18170.1	-	-	-	-	−3.5317	0.00243
WRKY DNA-binding protein 7	cassava4.1_011062m.g	AT4G24240.1	-	-	-	-	−1.83753	0.03388
**T200 - MAP kinase genes**								
Mitogen-activated protein kinase 3	cassava4.1_010219m.g	AT3G45640.1	−2.34215	0.00793	−2.34215	3.97E-06	−2.34215	5.24E-06
MAP kinase 15	cassava4.1_006140m.g	AT1G73670.1	−2.46879	0.00884	-	-	-	-
Mitogen-activated protein kinase kinase kinase 19	cassava4.1_020998m.g	AT5G67080.1	-	-	−3.57051	2.45E-05	-	-
Mitogen-activated protein kinase kinase kinase 9	cassava4.1_003834m.g	AT4G08480.1	-	-	−3.24143	5.04E-05	−3.24143	5.51E-06
Mitogen-activated protein kinase kinase kinase 15	cassava4.1_008711m.g	AT5G55090.1	-	-	−3.42293	2.97E-03	-	-
Mitogen-activated protein kinase kinase kinase 15	cassava4.1_030459m.g	AT5G55090.1	-	-	−2.24639	4.73E-03	−2.24639	7.81E-03
Mitogen-activated protein kinase kinase kinase 19	cassava4.1_010778m.g	AT5G67080.1	-	-	−2.73367	5.11E-03	-	-
Mitogen-activated protein kinase kinase kinase 19	cassava4.1_023447m.g	AT5G67080.1	-	-	−3.22549	3.21E-02	-	-
MAPK/ERK kinase kinase 1	cassava4.1_025838m.g	AT4G08500.1	-	-	−2.22549	4.27E-02	-	-
MAP kinase kinase 7	cassava4.1_028556m.g	AT1G18350.1	-	-	−3.01903	4.81E-02	-	-
**TME3 - MAP kinase genes**								
Mitogen-activated protein kinase kinase kinase 19	cassava4.1_020998m.g	AT5G67080.1	1.61609	1.45E-03	1.61609	0.034401	-	-
Mitogen-activated protein kinase kinase kinase 19	cassava4.1_010778m.g	AT5G67080.1	-	-	2.59734	0.027843	-	-
Mitogen-activated protein kinase kinase kinase 15	cassava4.1_026704m.g	AT5G55090.1	-	-	3.40470	0.012378	-	-
MAP kinase 4	cassava4.1_010005m.g	AT4G01370.1	-	-	1.64238	0.030164	-	-
MAP kinase kinase 9	cassava4.1_011965m.g	AT1G73500.1	-	-	1.68164	0.037617	-	-
**T200 - Phytohormone signalling genes**								
Ethylene responsive element binding factor 5	cassava4.1_012714m.g	AT5G47230.1	−2.60003	0.04990	−4.22549	2.937E-05	−2.50424	0.00652
Ethylene responsive element binding factor 5	cassava4.1_012714m.g	AT5G47230.1	-	-	−2.60003	2.94E-05	−2.6000	6.53E-03
Ethylene responsive element binding factor 1	cassava4.1_013138m.g	AT4G17500.1	-	-	−4.01903	3.42E-05	-	
Ethylene responsive element binding factor 6	cassava4.1_032473m.g	AT4G17490.1	-	-	−2.02756	7.71E-05		-
Ethylene responsive element binding factor 4	cassava4.1_015499m.g	AT3G15210.1	-	-	−3.10319	1.12E-04	−3.10318	1.07E-04
Ethylene responsive element binding factor 4	cassava4.1_014721m.g	AT3G15210.1	-	-	−2.80798	2.74E-03	−2.80798	6.10E-03
Ethylene responsive element binding factor 1	cassava4.1_022027m.g	AT4G17500.1	-	-	−3.10709	1.23E-03		
Ethylene response factor 7	cassava4.1_034303m.g	AT3G20310.1	-	-	-	-	1.86197	8.31E-03
erf domain protein 9	cassava4.1_032424m.g	AT5G44210.1	-	-	−2.17239	1.13E-05	−2.17239	2.63E-05
erf domain protein 9	cassava4.1_014544m.g	AT5G44210.1			−2.97522	1.81E-04	−2.97522	2.54E-04
Jasmonate-zim-domain protein 1	cassava4.1_014096m.g	AT1G19180.1	-	-	−2.27971	3.27E-03	-	-
Jasmonate-zim-domain protein 1	cassava4.1_013620m.g	AT1G19180.1	-	-	−2.21310	3.52E-03	-	
Jasmonate-zim-domain protein 8	cassava4.1_018315m.g	AT1G30135.1	-	-	−6.29587	1.07E-05	−6.29587	2.06E-02
Jasmonate-zim-domain protein 10	cassava4.1_017020m.g	AT5G13220.1	-	-	−2.40606	4.51E-03	-	-
Jasmonate-zim-domain protein 12	cassava4.1_015456m.g	AT5G20900.1	-	-	−2.12735	5.94E-03	−2.12735	2.85E-03
Jasmonate-zim-domain protein 3	cassava4.1_009349m.g	AT3G17860.1	-	-	−2.02736	6.81E-03	−2.02736	5.89E-03
Jasmonate-zim-domain protein 1	cassava4.1_031135m.g	AT1G19180.1	-	-	−3.19306	1.85E-02	-	-
Jasmonate-zim-domain protein 8	cassava4.1_019045m.g	AT1G30135.1	-	-	−3.01903	4.81E-02	-	-
Gibberellin-regulated family protein	cassava4.1_019648m.g	AT1G74670.1	-	-	3.13766	2.57E-04	3.13766	1.14E-02
Gibberellin-regulated family protein	cassava4.1_019838m.g	AT5G14920.1	-	-	3.71114	4.32E-04	3.71114	2.67E-03
Gibberellin-regulated family protein	cassava4.1_019810m.g	AT1G74670.1	-		2.09802	5.52E-04	2.09802	1.25E-04
Gibberellin-regulated family protein	cassava4.1_028672m.g	AT1G22690.2	-	-	2.06102	2.78E-03	-	-
Gibberellin 2-oxidase 8	cassava4.1_024994m.g	AT4G21200.3	-	-	3.89085	6.87E-03	-	-
Brassinosteroid-responsive RING-H2	cassava4.1_017699m.g	AT3G61460.1	-	-	−1.94589	1.70E-05	-	-
Auxin response factor 16	cassava4.1_002960m.g	AT4G30080.1	-	-	2.89517	9.36E-04	-	-
Auxin response factor 16	cassava4.1_009838m.g	AT4G30080.1	-	-	2.43627	8.52E-03	-	-
Auxin-responsive GH3 family protein	cassava4.1_004196m.g	AT4G03400.1			1.70739	2.98E-02	-	-
**TME3 - Phytohormone signalling genes**								
erf domain protein 9	cassava4.1_032424m.g	AT5G44210.1	−1.88098	1.82E-02	-	-	-	-
Ethylene responsive element binding factor 1	cassava4.1_013138m.g	AT4G17500.1	-	-	2.2302	0.003676	-	-
Ethylene response factor 1	cassava4.1_015673m.g	AT3G23240.1	-	-	2.01957	0.016286	-	-
Ethylene responsive element binding factor 4	cassava4.1_014721m.g	AT3G15210.1	-	-	-	-	−1.5327	0.040184
Jasmonate-zim-domain protein 1	cassava4.1_014096m.g	AT1G19180.1	−2.15968	0.00471	1.79727	4.71E-03	-	-
Jasmonate-zim-domain protein 1	cassava4.1_013620m.g	AT1G19180.1	-	-	2.42433	0.00506	-	--
Jasmonate-zim-domain protein 1	cassava4.1_031135m.g	AT1G19180.1	-	-	2.0092	0.02233	-	-
Jasmonate-zim-domain protein 8	cassava4.1_018315m.g	AT1G30135.1	1.62177	2.48E-02	1.62177	0.032334	-	-
Jasmonate-zim-domain protein 8	cassava4.1_019045m.g	AT1G30135.1	-	-	2.5862	0.007889	2.58620	0.031204
Jasmonate-zim-domain protein 8	cassava4.1_026855m.g	AT1G30135.1	-	-	3.31981	0.007962	-	--
Jasmonate-zim-domain protein 10	cassava4.1_016821m.g	AT5G13220.1	-	-	3.06848	0.000172	3.06848	0.034474
Jasmonate-zim-domain protein 12	cassava4.1_015456m.g	AT5G20900.1	-	-	1.64996	0.045744	-	-
Brassinosteroid-responsive RING-H2	cassava4.1_017695m.g	AT3G61460.1	−2.22022	3.82E-02	-	-	-	-
Brassinosteroid-responsive RING-H2	cassava4.1_018087m.g	AT3G61460.1	-	-	2.56082	0.003351	-	-

The identification and characterization of R genes has long been under scrutiny, where 7 major classes have been identified [79]. To date, research has focused on three dominant viral *R* genes, which includes the *Rx* gene against *Potato virus X* [80], *RT4-4* gene against *Cucumber mosaic virus* and *N* gene resistance against *Tobacco mosaic virus*. The identification in this study of fifteen TIR-NBS-LRR class I R genes, and presence of one represented CC-NBS-LRR (class II) gene in T200, is interesting in itself as it compares with previous cloned *Rx, RT4-4* and *N* resistance genes which also contain TIR domains. The down-regulation of TIR-NBS-LRR implies that TIR-NB-LRR receptor activation in cassava T200 is repressed and therefore SACMV may be avoiding detection and inhibition by plant defence response, therefore promoting virus replication and movement. Furthermore, suppression of TIR-NBS-LRR could negatively affect other signalling pathways downstream of TIR-activation such as the mitogen-activated protein kinase pathway. Collectively, the high number of repressed R genes at 32 and 67 dpi in T200 strongly supports a significant role in susceptibility to SACMV.

Resistance to CMD from wild-species such as *Manihot glaziovii* [81] was shown to be polygenic and recessive (designated CMD1), while in several African landraces, including TME3, additional sources of durable resistance were identified [9, 82], and were associated with a dominant R gene (CMD2) [10]. Subsequently, markers associated with the CMD2 trait were used in marker-assisted introgression of the gene into other genotypes [83] to understand its complementarity with CMD1, and results revealed that the landraces exhibit polygenic inheritance and that the genes are not linked and were non-allelic [84]. However despite these many studies, the genetics of resistance in cassava is not understood. In a recent study by Gedil et al. [85], they identified only 7 putative NBS-LRR R gene analogues from cDNA and DNA amplification in TME3 and surprisingly a higher number (35) in the highly susceptible landrace TME117. From this study, infectivity assays, virus load and transcriptome data for TME3 do not demonstrate early R gene-mediated responses in this landrace. Rather, results from this study point to a tolerance mechanism in TME3 as a result of highly suppressed transcripts at 12 dpi and mild symptoms (lower virus titres compared with T200), activation of some defence-related genes at 32 dpi, followed at 67 dpi by a recovery phenotype associated with a high number of repressed transcripts, thus creating an unfavourable cellular environment to support SACMV infection. Although cassava resistance genes *CMD1* and *CMD2* have been located on linkage maps of cassava, these genes have not yet been identified and mapped to any scaffolds of version 4.1 of the cassava draft genome presently available through phytozome, and therefore the potential role of these two genes in CMD resistance remains to be elucidated. In summary, the remarkable lack of R gene response in the tolerant TME3 landrace at 12 dpi, in comparison with the highly susceptible T200 where most R genes were down-regulated, and a notable up-regulation of eight R gene homologues at 32 and 67 dpi in TME3, support a role for these R genes in the recovery of TME3 to SACMV infection.

### Gene silencing

Previous studies, such as cassava infected with either *African cassava mosaic virus* (ACMV) or *Sri Lankan cassava mosaic virus* (SLCMV) [86], have shown that transcriptional (TGS) and post-transcriptional silencing (PTGS) is involved in recovered tissue [16], and these mechanisms may also play a simultaneous role in TME3 recovery. Geminiviral genome methylation has been shown to be an epigenetic defence response to geminiviruses [14, 87], and plant small RNAs play a role in biotic responses to plant virus pathogens (reviewed in [88, 89]). In recovered pepper leaves from *Pepper golden mosaic virus* (PepGMV), there was no difference between the number of differentially expressed genes between recovered and symptomatic leaves compared to mock-inoculated, and a higher number of genes were up-regulated compared to down-regulated. This was not the case in SACMV-infected TME3, where a high number of transcripts were repressed at 32 and 67 dpi. Within the set of altered defence response genes in pepper, there appeared to be little difference between recovered and symptomatic leaves, but rather a new set of genes were identified including genes involved in histone modification, supporting a role for TGS in recovery [15]. Several up-regulated histone superfamily proteins were identified in T200 at 12, 32 and 67 dpi, while histone 4 was highly expressed at 12 dpi, and less so at 67 dpi (Table 2). Histone family H2A7, 2A8 and 2A10 were also up-regulated in T200, while in TME3 only histone acetyltransferase of the MYST family1 was significantly down-regulated (2-fold, −3.176) at 67 dpi recovery. Histones play a role in chromatin structure, DNA replication and regulation of transcription, and in plants histone modification influences DNA methylation [90–92]. Histone H3 has been shown to be involved in geminivirus replication [93], while histones H2 and H4 (located in the golgi apparatus or cytosol) are involved in nucleosome assembly [94]. Up-regulation of histones 2A and 4 by SACMV indicates a role in replication, since geminiviruses form mini-chromosomes in the nucleus, while in TME3 there is no transcriptome evidence for up-regulation in response to SACMV. Histone modification by acetylation and methylation plays a role in regulation of transcription and cell-cycle regulation, and while the role of histone acetyltransferase (HAT) of the MYST family1 in cassava is not elucidated, down-regulation in TME3 suggests a putative role in counteracting cell-cycle dependent geminivirus replication [31]. In a similar study of SACMV-responsive transcripts in the susceptible host *Nicotiana benthamiana* [95], histone H3 (Log_2_ = 1.24 vs. Log_2_ = −1.22) and histone H4 (Log_2_ = 1.65 vs. Log_2_ = −1.76) were also found to be induced, while in recovered pepper leaves from PepGMV [15] these were repressed. The role of histone modification in plant geminivirus infection needs futher investigation.

To support a role for RNA silencing or methylation in the susceptible and tolerant phenotypes of T200 and TME3, respectively, NGS sequencing and quantification of small silencing RNA (vsRNA) populations (21–25 nt) targeting SACMV genomic DNA A and DNA B components in infected T200 vs. TME3 (at 12, 32 and 67 dpi) was performed (unpublished results). Normalized data revealed that the number of vsRNAs targeting SACMV DNA components in T200 was consistently higher compared with TME3. In both T200 and TME3 there was a significant increase in vsRNAs against DNA A and DNA B from 12 to 32 dpi despite persistence of symptoms and virus replication. However in T200 at 67 dpi there was a massive decrease in vsRNAs targeting DNA A and B, which led to a significant increase in virus replication and symptom severity, while in comparison, in TME3 the levels of vsRNAs increased, associated with a recovery phenotype (unpublished results). Although siRNA populations can range in length between 21- and 26 nt, the 24-nt siRNA range, produced by DCL3 [96, 97] cleavage, has primarily been associated with siRNA-mediated DNA methylation (RdDM). Notably, the 24 nt siRNA size class was the most highly represented amongst the siRNA populations targeting SACMV DNA A and B. The 24 nt siRNA populations targeting SACMV DNA A in T200 and TME3 declined from 12 to 32 dpi, but in contrast while the 24 nt siRNA population remained almost the same in T200 from 32 to 67 dpi, in the tolerant TME3 landrace the quantity increased significantly. In the case of DNA B in T200, the quantity of 24 nt siRNAs declined significantly from 12 to 32 dpi and remained almost at the same level at 67 dpi, likely promoting rapid virus movement since DNA B encodes movement functions. In comparison, in TME3 the 24 nt class of siRNAs, while remaining at a higher quantity compared to the other siRNA classes (21, 22, 23, 25 nts), did not change significantly across the course of infection.

Twelve methyl-CpG-binding domain proteins (MBD) have been identified and characterized in *Arabidopsis* and these function with chromatin remodelling proteins to inactivate gene expression and control chromatin structure mediated by CpG methylation [98, 99]. One unique observation made with TME3 at 67 dpi, but not at any other time points in T200, was the up-regulation of methyl-CpG-binding domain protein (MBD cassava4.1_028187m.g; Log_2_ = 2.478) which could bind to methylated CpG regions on SACMV DNA-A and B, therefore inhibiting replication. This could be one of the reasons accounting for lower viral titres and the recovery phenotype observed in TME3 at 67 dpi as compared with T200.

The recovery phenotype is observed in TME3 from ~55 dpi onwards (in this study sampled at 67 dpi), and we conclude that evidence collectively points to durable resistance or tolerance in TME3, mediated by concomitant early suppression of genes (likely to be involved in creating a supportive cellular environment for replication), persistent RNA silencing maintenance of genes required by SACMV as evidenced by a significantly lower number of altered transcripts throughout infection, and by methylation-associated TGS of SACMV DNA-A and B. This is also evident by a decline in virus load and symptoms at recovery. While in this study, there was little evidence for altered gene expression in RNA silencing associated transcripts such as *DCL*s, *RdRP*s or *AGO*s, in either T200 or TME3, Raja et al. 2008 [14] elegantly demonstrated that *Arabidopsis* mutants defective in a number of genes that are key players in the RdDM pathway (eg *drm1,drm2, kyp2*, *ago4* and others) results in hyper-susceptibility to infection with the geminiviruses *Cabbage leaf curl virus* (CaLCuV) and *Beet curly top virus* (BCTV).

### Differential expression of signalling, stress-related proteins, PR-proteins, WRKY transcription factors and MAP kinases

For biological processes, response to stress and biotic/abiotic stimuli were highly represented categories in both T200 and TME3 (Figure 3). Differentially expressed 2-fold genes were shown to be primarily transcription factors involved in basal immune or phytohormone signalling pathway activation and other metabolic processes, and many were similar to those reported in other biotic/virus-host interactions (reviewed in Whitham et al.) [18, 44]. An interesting observation revealed that of the 75 cassava T200 scaffolds involved in defence responses, approximately 68% were down-regulated. In addition to the disease resistance proteins discussed earlier, repressed transcripts observed included Ribonuclease P family protein (RPP1), Resistance to *P. syringae* pv. Maculicola 1 (RPM1), Mildew Resistance Locus O (MLO2, MLO12) and Non-host Resistance to P.S. Phaseolicola 1 (NHO1) resistance proteins; transcription factors such as WRKY; and heat shock proteins (HSPs) which are involved in defence (Additional files 3, 4, 5, 6, 7, 8, 9 and 10). In addition, transcripts such as MAPKs, and the signalling molecules ERF5 (ethylene responsive factor 5) and JAR1 involved in phytohormone signalling were also altered. Other signalling and regulatory proteins, such as calmodulin-binding proteins, that are involved in regulation of gene expression and signal transduction [100] were also significantly induced/repressed at different time points post infection. Calmodulin-like genes 23 (cassava4.1_017956m.g), calmodulin-like 37 (cassava4.1_029375.g) and calmodulin-like 42 (cassava4.1_016701m.g) were down-regulated in susceptible T200 at 32 (−3.6 log2 fold) and 67 (−2.8 log2 fold) dpi, but at 32 dpi, calmodulin-like 42 was induced in the tolerant cassava TME3 (Additional files 6, 7, 8, 9 and 10). It has been reported in many studies that calmodulin-like proteins are involved in defence and signalling against pathogen and insect attack and function in pathogen resistance [100]. Induction of calmodulin-like 42 at 32 dpi in TME3 indicates an appropriate defence response, while in T200 this is suppressed, leading to infection.

Transcript levels for two pathogenesis‒related protein (PRP) genes were shown to be increased upon infection by SACMV primarily at 32 and 67 dpi in T200 (Additional files 3, 4 and 5; Additional file 9), indicating a delayed immune response which persists even at full symptomatic infection. These PRPs included peroxidase (cassava4.1_011768m.g, cassava4.1_012124m.g) and thaumatin superfamily protein (cassava4.1_014480m.g, cassava4.1_014683m.g, cassava4.1_011211m.g). Log_2_ expression ratios ranged between 1.76 and 2.05 for peroxidase and between 2.28 and 3.59 for thaumatin. The induction of pathogenesis-related genes has been reported in other stress treatments and virus infections using gene expression tools [33, 100–103]. Despite induced basal defences in T200, these PRPs are not capable of inhibiting viral replication and spread, as demonstrated by the progressive increase in symptom severity, virus titre and high number of repressed genes over the infection period. It has been shown in many compatible plant virus-host studies, that despite progression of disease symptoms, some defence-related responses persist throughout the infection but have no effect on viral infection.

Studies in *Arabidopsis*, and several other plant hosts, have provided direct lines of evidence that some WRKY transcription factors (TFs) and MAP kinases are involved in plant defence response. The MAPK signalling pathway is evolutionary conserved, and MAP kinases primary role is to transfer sensors to cellular responses [104]. A MAPK signalling cascade is sequentially activated by three protein kinases, a MAP kinase kinase Kinase (MAPKKK or MEKK), a MAP kinase kinase (MAPKK or MKK) and a MAP kinase (MPK). Activation of this multi-tiered cascade is phosphorylation-dependent [105, 106]. Twenty MAPKs have been identified in *Arabidopsis* [107] where MAPK3, MAPK4 and MAPK6 in particular are stress/pathogen-responsive and have been the most comprehensively studied [108–110]. MAPK4 has been identified as important regulator in defence [31], and is a negative regulator of Salicylic acid (SA) signalling but a positive regulator of jasmonic acid (JA) signalling [111, 112]. In addition, MAPK3 and MAPK6 which are found downstream to MKK4/MKK5 have also been shown to regulate auxin and ROS signalling [27]. WRKY TF’s have been implicated in many stress-responses as fungal elicitors, pathogen responses, and in SA signalling [100]. A study by Liu et al. (2004) [113] demonstrated that virus-induced gene silencing of three WRKY genes (NtWRKY1, NtWRKY2 and NtWRKY3) in *Nicotiana tabacum* resulted in compromised N-gene-mediated resistance to *Tobacco mosaic virus.* Furthermore, *RRSI*, a gene that confers resistance to bacterial pathogen *Ralstonia solanacearum* encodes a TIR-NBB-LRR protein with a C-terminal WRKY motif (WRKY52). This additional WRKY structural feature of RRS1 could indicate a direct relationship between Avr-recognition and the downstream transcriptional activation of defence genes [114]. In this study, in addition to repression of R gene homologues, ten WRKY TFs and several MAPK signalling pathway genes (mitogen-activated protein kinase 3 (MAPK3), mitogen-activated protein kinase kinase kinase 15 and mitogen-activated protein kinase 9) were persistently down-regulated in T200 at 12, 32 and 67 dpi. Interrogation of the TME3 data at the same time points did not show any of the same patterns as T200 with regard the expression of WRKY and MAPK genes, however WRKY40 (cassava4.1_011696m.g) and MAPKKK19 (cassava4.1_020998m.g) were found to be upregulated in TME3 at 12 and 32 dpi, respectively. Amongst the suppressed WRKY transcripts in susceptible T200 at 32 and 67 dpi, were WRKY33 (cassava4.1_004465m.g), WRKY40 (cassava4.1_033249m.g), WRKY41 (cassava4.1_011518m.g) and WRKY70 (cassava4.1_012154m.g). Currently, eight WRKY TFs have been shown to be involved in defence in *Arabidopsis* [115]. AtWRKY18, AtWRKY38, AtWRKY53, AtWRKY54, AtWRKY 58, AtWRKY59, AtWRKY66 and AtWRKY70 were identified as targets for NPR1 which is an essential component in SA signalling. WRKY70, a positive regulator of SA-mediated defences while repressing JA signalling [105, 116], was down-regulated in susceptible cassava T200 at 67 dpi (Additional file 5). It is suggested that repression of this TF may contribute to suppression of the SA pathway, to subvert an induced resistance response in T200. Down-regulation of TFs and susceptibility in T200 is further supported by evidence of down-regulation of WRKY33 in T200, which may indirectly lead to inhibition of PHYTOALEXIN DEFICIENT 3 (PAD3), which is responsible for activating expression of antimicrobial camalexin. AtWRKY33 and MAPK4 form an indirect interaction with each other through the Map Kinase 4 Substrate 1 (MKS1) complex. MKS1 functions not only as an adaptor protein but has been shown to enhance the DNA-binding activity of AtWRKY33 [117]. Upon pathogen perception, a complex forms with MAPK4 (and its upstream kinases, MAKK1/MAKK2 and MEKK1), causing dissociation and release of WRKY33 and MKS1 from the complex, allowing for MKS1-AtWRKY33 to bind to the promoter region of PAD3. Co-suppression of associated MSK1-WRKY33 would prevent transcriptional activation of PAD3. Furthermore, geminivirus AC3 has also been shown to interact with host proteins such as DNA-J like proteins which are involved in protein folding and NAC transcription factors (NAC), which have been shown to regulate JA-induced expression [118]. Results from this SACMV-cassava study, support the hypothesis that concomitant suppression of NAC, WRKY, MAPK, and TIR-NBS-LRR transcripts in T200 leads to enhanced susceptibility, and that the disease phenotype is maintained with the avoidance of R-mediated resistance and/or other mechanisms. This correlates with viral quantification data showing increase in SACMV titre over the sixty-seven day period, as well as the increase in symptom severity over time. Furthermore, although the effect of MAPK-mediated phosphorylation on the function of WRKY remains to be defined, we also speculate that due to the down-regulation of MAPK3 (cassava4.1_010219m.g), reduced levels of MAPK3 leads to a reduction in phosphorylation of transcription factors such as WRKY which may directly be responsible for the down regulation of defence-related genes.

### Phytohormone signalling

Hormones, such as ethylene (ET), jasmonic acid (JA), abscissic acid, gibberellins and *s*alicylic acid (SA) are present in plants in basal amounts, yet act in a well-balanced and regulative manner during plant growth and development [119]. Any change from normal levels of phytohormones such as those caused by infection with virus pathogens could significantly alter physiological processes and morphology, resulting in symptoms such as stunting and leaf deformation, as was observed in our study. One striking observation for both T200 and TME3 across infection time points was the absence of altered genes that are reported to activate and regulate the SA signalling pathway such as ENHANCED DISEASE SUSCEPTIBILITY 1 (EDS1) and PHYTOALEXIN DEFICIENT 4 (PAD4), even though induction of transcription factors such as WRKY70 (cassava4.1_012154m.g) and WRKY33 (cassava4.1_007752m.g), and the PRP-3 (AT3G12500) marker gene, indicate some activity of the SA pathway early in infection. This is particularly interesting, especially for tolerant line TME3, as numerous studies have shown that SA plays an essential role in signal transduction pathways leading to the dramatic accumulation of pathogenesis-related (PR) transcripts culminating in a disease resistance response [120]. However in tolerance, such as demonstrated by TME3, SA does not play a major role in defence, as is the case in early induction of classical HR resistance. Rather, transcriptome results overall support preferred JA and ET responses over SA in both susceptible and tolerant cassava T200 and TME3. Suppression of jasmonate ZIM domain (JAZ) proteins in T200 and TME3 could lead to the activation of the JA pathway since JAZ1 (cassava4.1_013620m.g), JAZ8 (cassava4.1_019045m.g) and JAZ12 (cassava4.1_015456m.g) are differentially expressed (Additional file 9 and Additional file 10). In cassava T200, JAZ1, JAZ8, and JAZ12 exhibited down-regulation at 32 dpi and/or 67 dpi, whereas in tolerant TME3, JAZ1 and JAZ8 were up-regulated at 12 dpi, but down-regulated at 32 and/or 67 dpi. In addition, JAZ12 was also repressed in TME3 at 32 dpi. The down-regulation of JAZ could possibly be attributed to the SCF (Skp1-Cullin-F-box) complex which mediates the degradation of JAZ proteins, and in turn leads to relieve JA repression [121, 122]. JAZ proteins are involved in a negative regulatory feedback loop with MYC2 transcription factors (reviewed in Chico et al.) [123]. In brief, under normal conditions, JAZ proteins act as repressors by binding to MYC2 thereby inhibiting the transcription of early JA-responsive genes. Therefore, with the response to stimulus, such as pathogen attack, JA activation will be mediated by 26S proteasome degradation of JAZ repressors that consequently releases MYC2, allowing for downstream transcriptional activation of JA. The suppression of JAZ in the T200 in response to SACMV suggests that lower levels of JAZ are available for repression of MYC2, thereby allowing the transcription of downstream defence – responsive genes. Furthermore, lipoxygenase (cassava4.1_001238m.g), involved in the early steps in JA synthesis, was also found to be down-regulated, and WRKY70, a repressor of JA signalling [103, 116], was down-regulated in susceptible cassava T200 at 67 dpi, further supporting a role in promoting SACMV infection. Pierce and Rey, 2013 [47] also reported that JA signalling pathway responses were favoured over SA signalling in the *Arabidopsis*-SACMV interaction study, since marker genes for JA were more prevalent and highly expressed throughout the course of infection compared to SA.

ET is influential in mediating the outcome of synergism or antagonism between JA and SA signalling. ET is able to bypass key regulator genes such as NPR1 in SA signalling during SA/JA crosstalk therefore preventing suppression of JA signalling [121, 122]. ET and JA pathways, in many instances, have been shown to regulate similar type of defence genes [46, 124]. Ethylene-responsive element binding factors (ERF) proteins are plant-specific transcription factors that respond to ET signalling [125] which may be altered by pathogen infection [126, 127], and play important roles in plant responses to various hormones or environmental changes. For example, the induction of ERFs following infection by viral pathogens such as *Tobacco mosaic virus* [126] has been demonstrated. Repression of several ERFs, such as ERF-5 (cassava4.1_012714m.g), ERF-9 (cassava4.1_014544m.g) and ERF-4 (cassava4.1_014721m.g) (Additional file 9) was evident at 12, 32, and 67 dpi in cassava T200. In contrast, for TME3, no ethylene-responsive element binding factors were found to be significantly changed across any of the three timepoints, again supporting the collective evidence for other tolerant-related mechanisms in TME3. Results for T200 suggest that SACMV infection is promoted by negative regulation of ERFs and lack of host elicitation of SA pathway-dependent defence, which reduces the defence reponse. A report by Love et al. [127] showed that ethylene-signalling mutants reduced virus titers of *Cauliflower mosaic virus* and hindered long-distance movement of the virus. SACMV infection in cassava T200 appears to be supported by evasion of basal host defence via overall negative regulation of JA and ET signaling pathways and lack of host elicitation of SA pathway dependent resistance.

Gibberellin-regulated family proteins (cassava4.1_019648m.g, cassava 4.1_019838m.g, cassava4.1_019810m.g, cassava4.1_028672m.g and cassava4.1_024994m.g) (Additional files 1, 4 and 5; Additional file 9) were consistently up-regulated in T200 plants, particularly at 32 and 67 dpi, and although the role of gibberellins in cassava is not clear, they may play a role in symptom phenotype.

Comparisons between our data and that of Miozzi and collegues [48] indicates that there are striking differences in the the phytohormone signalling pathways changed during TYLCSV infection in tomato, in relation to SACMV infection in cassava. While we observed expression changes primarily of genes involved in the JA and ET signalling pathways, TYLCSV was reported to primarily cause changes in the expression of genes involved in the gibberrellin and abscisic acid pathways. The differences in expression between TYLCSV and SACMV indicate that the role of phytohormone signalling in geminvirus-plant interactions is variable and complex, and is host-pathogen dependent. Furthermore, the difference observed in phytohormone responses may also be attributed to the types of cells and tissues infected by TYLCSV (a phloem-limited virus restricted to cells of the vascular system) and SACMV (a non-phloem limited virus which invades mesophyll tissue).

### Changes in cell wall and plasmodesmata-associated genes

The plasmamembrane component was highly represented in T200 and TME3, and there was also a noticeable expression of cell wall-related transcripts (Figure 3). In a study by Shimizu et al. [128], it was reported that *Rice dwarf virus* infection in rice plants resulted in the repression of several cell-wall related genes. This cassava transcriptome study revealed that the opposite was true for susceptible T200 infected with SACMV. The up-regulation of several host genes that encode for cell-wall polysaccharides, and enhanced expression of plasmodesmata-associated genes, particularly at heightened infection at 32 dpi and 67 dpi (Additional file 4 and Additional file 5; Additional file 9), suggested a role in SACMV movement. The same genes were not detected in tolerant cultivar TME3 at either time point. These genes include, plant invertase (cassava4.1_016774m.g, cassava4.1_021617m.g), cellulose synthase (cassava4.1_001280m.g), pectin methylesterase (cassava4.1_004357m.g), pectin lyase (cassava4.1_005619m.g, cassava4.1_007568m.g, cassava4.1_009002m.g), β-tubulin (cassava4.1_007617m.g, cassava4.1_007632m.g), expansin (cassava4.1_014066m.g, cassava4.1_014407m.g, cassava4.1_014440m.g, cassava4.1_014489m.g), plasmodesmata callose-binding protein 3 (cassava4.1_016458m.g, cassava4.1_016746m.g), calreticulin (cassava4.1_008376m.g) and arabinogalactan protein (cassava4.1_018722m.g, cassava4.1_029618m.g). The induction of these genes firstly suggests that there may be a large number of cell wall and plasmodesmata modifications that occur within infected cells, but whether these modifications are favourable to the virus is yet to be determined. However, what is true for virus infections, whether in compatible or incompatible interactions, is the increase in nutrient demands of the host as well as the cellular demands of mounting a defence response. The enhanced expression and activity of cell wall invertases for example and its role as in plant-pathogen interactions has been reported in several studies [129–133]. Several lines of evidence indicate that an increase in cell-wall invertase will result in the cleavage of sucrose into glucose and fructose which serve as the energy molecules that fulfill the carbon and energy demand of mounting a defence response against the invading pathogen [133, 134]. In addition, sugars such as glucose and sucrose serve as signalling molecules [135] which will prime the activation of PR genes following infection [136]. Furthermore, infection of tobacco plants with PVY showed sugar accumulation which was accompanied by an accumulation of transcripts encoding PR proteins [137]. Based on these results it was proposed that sugars act as amplifiers for plant defence responses during plant pathogen interaction [137]. Our study shows an up-regulation of invertase at the late stages of infection suggesting that the breakdown of sucrose could play a role in both the energy source and signalling molecules for impending defence responses against SACMV.

Also observed in our transcriptome data were the up-regulation of β-tubulin, pectin methylesterase (PME), calreticulin and plasmodesmata-callose binding protein. A number of previous studies have implicated a number of cellular components and proteins that are localised to the plasmodesmata (PD) and that play a role in either cell-to-cell communication or movement of molecules across the PD [138]. SACMV is a bipartite virus that has a DNA-B component harbouring two movement genes (BV1 and BC1) that encode movement proteins that act in a cooperative fashion to facilitate local and systemic movement of the virus. Despite, the presence of these movement proteins, the virus is still likely to require a number of host factors in order to aid its movement throughout the host plant. In a number of studies conducted, it has already been suggested that the viral movement proteins modify the PD and alter the plasmodesmal size exclusion limits (SEL) to allow the movement of viral protein–nucleic acid complexes to neighbouring cells [139–141]. Furthermore, the interaction between viral movement proteins, the PD and the host cytoskeleton has already been scrutinised for many virus-plant systems [142–148]. Pectin is enriched around the PD, and PME is an enzyme involved in pectin de-esterification, and has been shown to interact with virus movement proteins [149, 150]. It has been hypothesised that PME may act as a receptor protein which may be hijacked by plant viruses to aid in cell to cell movement. For example, PME has been shown to interact with TMV movement protein which assists the virus with cell-to-cell movement during infection [149, 150]. Chen et al., [148] further demonstrated through a yeast two-hybrid system, that the MPs from two other plant viruses, *Cauliflower mosaic virus* and *Turnip vein clearing virus* also bind to PME. We therefore speculate, that the induction of PD-associated genes in T200 is favouring cell-to-cell movement of the virus which has can be linked to the increase of SACMV titres observed at 32 and 67 dpi.

## Conclusions

**Figure 5:**
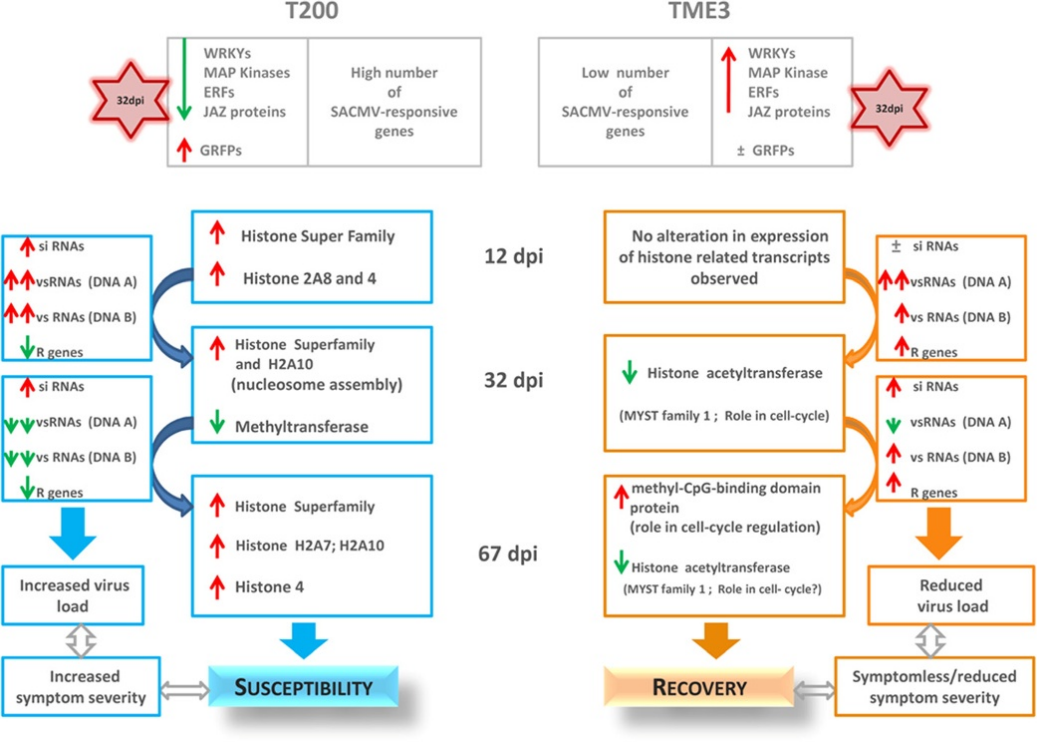
**Schematic model comparing some signalling molecules and pathways, activated in SACMV-challenged susceptible T200 and tolerant TME3, which may contribute, in addition to other interlinked factors, to a susceptible and tolerant phenotype, respectively.**

This is the first virus-responsive transcriptome study in cassava following the infection of a cassava geminivirus over three time points post infection, and it will prove interesting to compare these results in future with cassava in response to other pathogens, such as the bacterial pathogen *Xanthomonas axonopodis* pv. manihotis [68, 151]. Comparative transcriptome analyses of T200 and TME3 landraces revealed that many of the responses to SACMV infection were consistent with changes seen in other plants under biotic stress, but many were specific to the SACMV-cassava interaction. One of the most significant findings was that the number of transcriptome alterations induced by SACMV in TME3 was significantly lower compared with T200, and also in comparison with CaLCuV and SACMV in the susceptible host, *Arabidopsis* [31, 47], and may, in part, explain the recovery phenotype at 67 dpi observed in infected TME3 cassava leaves but not in susceptible T200. Additionally, what clearly emerged from our data, was that susceptibility in T200 is largely mediated by significant levels of transcriptome repression, rather than induction. Also, a particularly important result for T200 was the repression of many R-gene homologues throughout infection, providing strong evidence for a role in susceptibility. Equally interesting, repression of R gene homologues genes was not observed early in infected TME3 plants, but rather up-regulation of 8 and 2 R genes at 32 and 67 dpi, respectively, correlating with the recovery phenotype. Based on the results obtained in this study, and on available literature with regard to host-virus responsive genes, a comparative model of some possible responses contributing towards a tolerance and susceptible in T200 and TME3 is depicted in Figure 5. This model by no means suggests that these are the sole factors, and on the contrary, host-geminivirus interactions are known to involve complex interactive neworks. It is also important to take into account that cassava is a perennial crop and these changes in transcription due to virus infection are likely to be modulated throughout the life cycle of the plant. It would be interesting to follow these patterns over longer periods of time, as most NGS plant virus studies have focused on early time points of infection in annual crops such as tomato, *Arabidopsis* and tobacco. Additional analysis of the phylogenetic relationship between cassava TIR-NBS-LRR domains, and *Arabidopsis*, rice, castor bean, tomato and other plant species, is ongoing in our laboratory and will also prove interesting. Homology between these genes could provide some insight into the evolutionary conservation of these R genes.

In summary, CMD is a devastating disease caused by at least nine species of *Begomovirus*, and several species, including SACMV, have been identified in regions of South Africa and some neighbouring countries including Zimbabwe, Mozambique and Swaziland. Understanding the mechanisms underlying CMD could facilitate control strategies to combat begomoviruses, either through genetic modification approaches or through breeding programs, which could result in conferring resistance or a degree of tolerance. The knowledge from this study will serve as a useful genetic resource for relevant cassava researchers globally. A systems biology approach is required to build geminivirus-interaction models, and complementary studies on small RNA population responses in T200 and TME3 (have been completed but is not the remit of this study), and further gene identification and verification of candidate gene functions, can lead to achieving this goal. Additional metabolome and proteome data will in future be needed to develop a comprehensive interactome model for geminivirus infection in host plants.

## Methods

### Micro-propagation and acclimatization of cassava

Cassava T200 and TME3 landraces were micro-propagated by nodal cutting culture on Murashige and Skoog (MS) medium [152] supplemented with 20 g/L sucrose and 7.8 g/L plant agar (Sigma Aldrich), pH 5.8. Cassava explants were allowed to grow at 25°C under a 16 hour photoperiod at a light intensity of 150 μEm^−2^ sec^−1^. At the appearance of roots (approximately 10 days), plantlets were transferred into Jiffy® pellets (Jiffy Products International) which were placed on a tray that was covered with plastic film and placed in a controlled growth chamber (28°C; 16 hour photoperiod). Plantlets were gradually acclimatized by adding slits to plastic film. Acclimatized plantlets were allowed to grow until they reached a 4–6 leaf stage.

### Agroinoculation of T200 and TME3 plantlets

Agroinoculation of T200 and TME3 cassava plantlets was achieved by a protocol adapted from Hayes et al. [153]. Infectious, head-to-tail, dimers of SACMV DNA-A and DNA-B were previously cloned separately into binary vector pBIN19 [7] and transformed into *Agrobacterium tumefaciens Agl*. The two transformed cultures containing DNA-A and DNA-B were cultured separately in Luria Bertani (LB) Broth supplemented with carbenicillin (100 μg.ml^−1^) and kanamycin (100 μg.ml^−1^). Wild-type *Agrobacterium Agl*1 cultures served as a negative control for inoculations and was inoculated into LB broth supplemented with carbenicillin (100 μg ml^−1^). Cultures were grown overnight at 30°C until optical densities of 1.8-2.0 (OD_600_) were reached. From each of the three cultures, 5 ml was sub-inoculated into 30 ml fresh LB Broth, containing the correct combination of antibiotics as previously described. Cultures were once again grown overnight at 30°C until cultures reached optical densities of 1.8-2.0 (OD_600_). For each culture, 25 ml aliquots were pelleted by centrifugation at 13000xg, washed in sterile distilled water and subsequently resuspended in 5 ml LB Broth. *Agl*1-SACMV DNA-A and *Agl*1-SACMV DNA-B were resuspended and combined to form a homogenous mixture of *Agl*1- SACMV DNA-A and *Agl*1- SACMV DNA-B cells. T200 and TME3 plantlets were wounded along the stem with a hypodermic needle and each plantlet was inoculated with 100 μl the *Agl*1DNA-A/DNA-B suspension using a 1 ml Hamilton syringe. Control plants were mock-inoculated with 100 μl wild-type untransformed *Agrobacterium Agl*1inoculum.

### Sample collection

SACMV-infected and mock-inoculated plants were monitored over a 67 day period. Newly developed symptomatic leaf tissue from apical leaves was collected from each plant (n = 6) at each time point i.e. 12, 32 and 67 dpi, and pooled. Leaves 2–3 under the apex were selected as geminiviruses are known to replicate in actively dividing cells [31]. Time points were however kept separate and therefore a total of six SACMV-infected samples were used in downstream sequencing (12, 32 and 67 dpi for T200 and 12, 32 and 67 dpi for TME3). The same procedure was carried out on mock-inoculated leaf tissue at the same time points therefore resulting in six samples of mock-inoculated controls. One gram of leaf tissue was immediately frozen in liquid and stored at −80°C until further use for DNA and RNA extractions.

### DNA extraction from leaf tissue

For each time point (12, 32 and 67 dpi), the leaves closest to the apex were harvested from six plants. Total nucleic acid (TNA) was isolated from these SACMV infected and mock-inoculated leaves using a modified CTAB-based extraction method [154]. Fifty milligrams of fresh leaf tissue was homogenized in liquid nitrogen. The resulting tissue powder was suspended in 500 μl of CTAB extraction buffer (2% CTAB, 1.4 M NaCl, 20 mM EDTA, 0.1 M Tris–HCl, pH 8.0). One μl of 2-mercaptoethanol was added to the suspension, which was incubated at 65°C for 1 h. The suspension was then purified twice by a chloroform: isoamyl alcohol (24:1) solution and precipitated with isopropanol. The TNA was recovered at ~13000 g at 4°C for 10 min. Recovered TNA pellets were washed in 70% ice-cold ethanol and later resuspended in TE buffer (10 mM Tris–HCl, 1 mM EDTA, pH 7.5) as well as treated with 1 μl of RNAse A (10 mg/ml) overnight at 4°C. The purity of the TNA was assessed using the NanoDrop™ ND-100 Spectrophotometer (NanoDrop Technologies, Thermo Scientific, USA).

### Confirmation of SACMV infection using conventional PCR

Systemic infection in cassava leaf tissue for T200 and TME3 at 12, 32 and 67 dpi was confirmed by conventional PCR. 50 μl PCR reaction were set up and contained 0.4 μM of each primer, 200 μM dNTPs, 2 units DreamTaq DNA polymerase (Fermentas, Vilnius, Lithuania), 1x DreamTaq Buffer (Fermentas,Vilnius, Lithuania), and nuclease-free water to a final volume of 50 μl. A 550 bp fragment of the core coat protein (CCP) on SACMV DNA-A was amplified using degenerate forward primer: (V524) 5′ GCCHATRTAYAGRAAGCCMAGRAT 3′ and reverse primer: (C1048) 5′ GGRTTDGARGCATGHGTACANGCC 3′. Approximately 500 ng of the total nucleic acid (TNA) template was added to the reaction mixture. Reactions were cycled in a MyCycler™ Thermal Cycler (Bio-Rad) at 95°C for 5 minutes to activate the Taq DNA polymerase, followed by 35 cycles of denaturation at 95°C for 30 seconds, annealing at 55°C for 30 seconds, primer extension at 72°C for 45 seconds, and a final extension step of 72°C for 5 minutes. DNA-A of SACMV cloned into pBluescript vector (50 ng) was used as positive control for PCR reactions. Amplification products were examined by electrophoresis on a 1.2% agarose TAE gel containing 10 μg/ml ethidium bromide.

### Real-time quantitative PCR of SACMV DNA-A

Determination of the viral titre in T200 and TME3 plants was achieved by use of qPCR on TNA extracted from both cultivars at time points 12, 32 and 67 dpi. TNA samples was all standardised to a concentration of 100 ng/μl. Duplicates of each sample were prepared as well as a no template control (NTC) of nuclease-free water. For each sample, a 20 μl reaction was set up in LightCycler capillaries containing 1 μl of 100 ng of leaf tissue TNA was added to 4 μl LightCycler ® FastStart DNA Master^Plus^ SYBR Green I (Roche), 1 μl forward coat protein primer (10 μM) 5′ACGTCCGTCGCAAGTACGAT3′, 1 μl reverse coat protien primer (10 μM) 5′ATTGTCATGTCGAATAGTACG 3′ and 14 μl nuclease-free water. A 150 bp fragment was amplified and quantified using the following amplification conditions: 95°C for 10 min, followed by 35 cycles of 95°C for 10 sec, 60°C for 10 sec, and 72°C for 15 sec. A single fluorescence measurement was taken at the end of each extension step during the PCR amplification cycle. A melting curve (65°C-95°C) with a heating ramp rate of 0.1°C/s and a continuous fluorescence measurement was conducted after the amplification and quantification cycle. A 166 bp PCR product of ubiquitin was amplified from 100 ng of the same TNA samples used for viral quantification which served as an internal loading control. Primers used were previously tested in cassava. Primer sequences used were UBQ10 (fwd): 5′ TGCATCTCGTTCTCCGATTG 3′ and UBQ10 (rev): 5′ GCGAAGATCAGTCGTTGTTGG 3′ previously described in Moreno et al. [155]. Data were exported to Microsoft Excel for statistical data analyses using the Students *t*-test.

### RNA extractions

Total RNA was extracted on SACMV-infected and mock-inoculated leaf tissue using a modified high molecular weight polyethylene glycol (HMW-PEG) protocol [156]. One gram of leaf tissue, for each biological replicate, was homogenised in liquid nitrogen and added to 5 ml preheated (65°C) GHCL buffer (6.5 M guanidium hydrochloride, 100 mM Tris–HCl pH 8.0, 0.1 M sodium acetate pH 5.5, 0.1 M β-mercaptoethanol) and 0.1 g HMW-PEG (Mr: 20 000, Sigma). The mixture was then pelleted by centrifugation (10000xg) for 10 minutes at 4°C. The supernatant was treated with 0.1 ml 1 M sodium citrate (pH 4.0), 0.2 ml 2 M NaCl and 5 ml phenol:chloroform:isoamyl acohol (PCI) (25:24:1). The mixture was then vortexed vigorously and again pelleted by centrifugation (10000xg) for 10 minutes at 4°C. The supernatant was removed and RNA was precipitated by adding 5 ml isopropanol (Sigma). The mixture was thoroughly mixed and incubated at −20°C for 60 minutes and pelleted by centrifugation (10000xg*)* for 25 minutes at 4°C. RNA pellets were washed with 5 ml ice-cold 75% ethanol. RNA Pellets were dried at 37°C for 5 minutes. The pellet was resuspended in 100 μl preheated (55°C) RNase-free water and 1 μl RNase inhibitor (Fermentas). Concentrations were determined using the NanoDrop™ 1000 spectrophotometer (Thermo Scientific, USA) and RNA integrity was assessed using an Agilent 2100 Bioanalyzer.

### cDNA library preparation and sequencing

cDNA libraries were generated at the Functional Genomics Center UNI ETH Zurich, Switzerland. Briefly, 12 ug of total RNA for each sample was used to generate cDNA libraries. RNA was fragmented and subjected to hybridization and ligation using the SOLiD Total RNA-Seq Kit (Applied Biosystems) according to the manufacturer’s instructions. cDNAs were selected by size on a polyacrylamide gel before and after the library amplification. A total of 12 libraries were multiplexed using the SOLiD RNA Barcoding Kit (Applied Biosystems) and pooled in an equimolar ratio. The samples were then diluted and used for emulsion PCR. Beads containing a multiplex of 12 samples were deposited onto a single flow cell. Libraries were sequenced operating on 50 bp forward and 35 bp reverse paired-end sequencing chemistry on the ABI SOLiD V4 system.

### Bioinformatics: assembly, mapping and annotation

The SOLiD v4 sequencer was used for the generation of sequence reads and was run in paired-end mode (50 + 35 bp). For each time point, differential gene expression data was achieved by normalization against mock-inoculated. This resulted in two csfasta and two quality files per sample. The reads generated for each library were mapped to the genome assembly (http://www.phytozome.net/cassava.php, *Manihot esculenta* 147, version 4.1) using the Lifescope software from LifeTech. As a result, SAM/BAM alignment files were prepared, sorted and indexed using samtools (http://samtools.sourceforge.net/). In the secondary data analysis phase, the BAM data were matched with the genome annotations available in Phytozome as a GTF/GFF3 file, which describes genes, transcripts and their exons with the genomes coordinates. The alignments were then transformed to counts using rnaSeqMap library (v.2.7.12) of Bioconductor [157] (release version 2.8). The count table for all genes from the annotation were analyzed using DESeq (v1.4.1) [158] from the same Bioconductor release. The procedure of finding significant expression regions was also performed for intergenic spaces, to find the probable regions of novel transcription, not known by the curators of the annotations in Phytozome. In order to identify and quantify the number of differentially expressed genes common between time points 12, 32 and 67 dpi in each landrace, data was imported into SQL 2012 where ‘inner join’ and ‘left join” queries were executed using the cassava transcript ID number as the unique feature used to identify all of the genes common between time points. Transcripts were filtered by applying a log_2_-fold cut-off with a p-value of <0.05 to select for highly expressed transcripts.

### RT-qPCR validations for genes differentially expressed in T200 and TME3

Fifteen genes (12 from T200 and 3 from TME3) that were found to be differentially expressed were selected based on the SOLiD RNA-seq results (i.e. >2- fold change, p < 0.05) and analysed using real-time quantitative RT-PCR. One of the criteria used to select genes, was the differential expression observed in at least 2 of the 3 time points in T200 and TME3 SACMV-infected leaf tissue. Primers for each gene were designed using software available online through Integrated DNA technologies (IDT, http://www.idtdna.com/Primerquest/Home/Index). In brief, 1 μg of DNase-treated total RNA was reverse transcribed using the Improm-II-reverse transcriptase kit (Promega, Madison, WI) according to manufacturer’s instruction. RNA, dNTPs and Oligo dT18 primer were denatured for 10 min at 70°C; then kept at 25°C for 5 min before the reverse transcription master mix was added. Reverse transcription was performed at 42°C for 1 hour followed by a 10 min incubation step at 70°C. Control reactions were set up without the addition of reverse transcriptase and used as negative controls in the real-time PCR study. RT-qPCR experiments were conducted on the Lightcycler 1.5 for all genes using the appropriate primer pair for each reaction (Additional file 14). Relative quantification standard curve method [71] was used to calculate the relative expression changes in each of the 8 genes assessed. Standard curves were generated for each gene using a 10-fold serial dilution of cDNA reverse transcribed from RNA extracted from either healthy T200 or TME3 leaf tissue. All reactions were based on the following recommended protocol using 0.5 μl of each primer and 1 μl of template per reaction. In brief, all qPCR reactions were performed in LightCycler® capillaries using the LightCycler 1.5 using LightCycler® FastStart DNA MasterPlus SYBR Green I kit (Roche). Three biological replicates and two technical replicate were run for SACMV-infected and mock-inoculated leave cDNA samples for T200 or TME3 at 12, 32 and 67 dpi. One μl of undiluted cDNA was used for each reaction. The cycling conditions used were as follows: initial denaturation for 10 min at 95°C (hot start) followed by an amplification and quantification cycle repeated 35 times, each consisting of 10 sec denaturing at 95°C, 10 sec annealing at primer specific temperatures, 15 sec primer extension at 72°C with a single fluorescence measurement. Melting curve cycle was obtained by heating to 65°C for 15 s with a heating rate of 0.1°C per second with a continuous fluorescence measurement. UBQ10 [158] was the gene used as an endogenous control for normalization. Statistical analysis was carried out in Microscoft Excel using the Students *t*-test.

### Availability of supporting data

The BAM sequence data sets supporting the results of this article have been curated and are available in the NCBI Sequence Read Achive (SRA). These files can be accessed using BioProject accession: PRJNA255198 [70] [http://www.ncbi.nlm.nih.gov/sra/?term=PRJNA255198]. Twelve experiment files are available under this Bioproject representing each library described in the manuscript. The experiment accession numbers are sequencial and range from SRX671492 to SRX671503. Furthermore, additional files supporting the results of this article have been uploaded to LabAchvives; these files are available using the DOI: 10.6070/H4028PGQ.

## Electronic supplementary material

Additional file 1: **Pairing statistics for cassava F3 and F5 Tags.** (DOCX 15 KB)

Additional file 2: ***Manihot esculenta −*** **147- annotated transcriptome_genes.** (XLSX 2 MB)

Additional file 3: **List of all differentially expressed genes in T200 at 12 dpi.** (XLSX 6 MB)

Additional file 4: **List of all differentially expressed genes in T200 at 32 dpi.** (XLSX 6 MB)

Additional file 5: **List of all differentially expressed genes in T200 at 67 dpi.** (XLSX 4 MB)

Additional file 6: **List of all differentially expressed genes in TME3 at 12 dpi.** (XLSX 6 MB)

Additional file 7: **List of all differentially expressed genes in TME3 at 32 dpi.** (XLSX 6 MB)

Additional file 8: **List of all differentially expressed genes in TME3 at 67 dpi.** (XLSX 6 MB)

Additional file 9: **Comparisons of number of differentially expressed genes between 12, 32 and 67 dpi in T200.** (XLSX 468 KB)

Additional file 10: **Comparisons of number of differentially expressed genes between 12, 32 and 67 dpi in TME3.** (XLSX 103 KB)

Additional file 11: **Transcript quantification for mock-inoculated T200 and TME3 leaf tissue.** (XLSX 82 KB)

Additional file 12: **Comparative analyses of Kegg metabolic pathways differentially expressed in SACMV-infected Arabidopsis, cassava T200 and TME3.** (XLSX 18 KB)

Additional file 13: **SACMV-responsive R gene homologues and histone-related genes in T200 and TME3.** (XLSX 11 MB)

Additional file 14: **Primers used for qPCR validations.** (DOCX 13 KB)

## Abbreviations

DEG: Differentially expressed genes
dpi: Days post infection
CMD: Cassava mosaic disease
ET: Ethylene
HR: Hypersensitive response
JA: Jasmonic acid
NGS: Next generation sequencing
R: Resistant
SACMV: South African cassava mosaic virus
SA: Salicylic acid
S: Susceptible
TF: Transcription factor.
