# Supplementary material for: Transcriptional analysis of South African cassava mosaic virus-infected susceptible and tolerant landraces of cassava highlights differences in resistance, basal defense and cell wall associated genes during infection
Source: BMC Genomics. 2014 Nov 20;15:1006. doi: 10.1186/1471-2164-15-1006 (PMC4253015; doi:10.1186/1471-2164-15-1006)
Supplement: Supplementary file 1 — Additional file 1: Pairing statistics for cassava F3 and F5 Tags. (DOCX 15 KB) [file 12864_2014_6706_MOESM1_ESM.docx]

**Additional File 1**: **Pairing statistics for the total number of bases and forward and reverse tagged sequences that were mapped to the cassava draft genome (version 4.1) for T200 and TME3 libraries.**

|  | T200 infected 12dpi | T200 Infected 32dpi | T200 infected 67dpi | T200 mock-inoculated 12dpi | T200 Mock- inoculated 32dpi | T200 Mock-inoculated 67dpi | TME3 infected 12dpi | TME3 Infected 32dpi | TME3 infected 67dpi | TME3  Mock-inoculated 12dpi | TME3 Mock- inoculated 32dpi | TME3  Mock-inoculated 67dpi |
| --- | --- | --- | --- | --- | --- | --- | --- | --- | --- | --- | --- | --- |
| Total number of bases in paired reads | 748,005,833 | 312,918,869 | 484,858,536 | 607,626,022 | 534,428,823 | 633,895,179 | 634,953,645 | 567,510,203 | 878,731,651 | 766,608,396 | 651,492,969 | 787,945,631 |
| F3 and F5 mapped (%) | 32.16 | 11.28 | 20.32 | 33.44 | 18.8 | 26.79 | 22.06 | 22.91 | 31.5 | 18.4 | 21.28 | 25.28 |
| F3 mapped, F5 unmapped (%) | 22.87 | 22.99 | 22.57 | 21 | 21.66 | 22.02 | 23.23 | 22.87 | 22.84 | 21.65 | 22.86 | 22.99 |
| F3 unmapped, F5 mapped (%) | 5.82 | 6.9 | 7.19 | 4.19 | 5.15 | 6.23 | 7.27 | 6.32 | 6.18 | 6.5 | 6 | 6.71 |
| F3 and F5 unmapped (%) | 39.15 | 58.83 | 49.93 | 41.38 | 54.39 | 44.96 | 47.44 | 47.89 | 39.48 | 53.43 | 49.86 | 45.01 |
| Total count of mapped reads |  |  |  |  |  |  |  |  |  |  | 215,499,20 | 249,149,44 |
|  | 200,801,05 | 163,420,84 | 167,942,02 | 150,030,93 | 184,254,97 | 183,472,80 | 217,820.32 | 182,493,03 | 238,740,00 | 270,717,82 |  |  |
|  |  |  |  |  |  |  |  |  |  |  |  |  |
| Total reads mapped (%) | 60.85 | 41.17 | 50.08 | 58.63 | 45.61 | 55.04 | 52.56 | 52.1 | 60.52 | 46.65 | 50.14 | 54.89 |
