# Supplementary material for: Transcriptional analysis of South African cassava mosaic virus-infected susceptible and tolerant landraces of cassava highlights differences in resistance, basal defense and cell wall associated genes during infection
Source: BMC Genomics. 2014 Nov 20;15:1006. doi: 10.1186/1471-2164-15-1006 (PMC4253015; doi:10.1186/1471-2164-15-1006)
Supplement: Supplementary file 14 — Additional file 14: Primers used for qPCR validations. (DOCX 13 KB) [file 12864_2014_6706_MOESM14_ESM.docx]

**Additional file 14: PCR primers used in real-time quantitative RT-PCR to verify selected differentially expressed transcripts in cassava T200 and TME3.**

| **Gene Name** | | **Phytozome Accession number** | | **Forward primer (5’-3’)** | | **Reverse primer (5’-3’)** | |
| --- | --- | --- | --- | --- | --- | --- | --- |
| Cellulose synthase | cassava4.1_001280m.g | | AGCAGCTGAAGGAACTTGGAGACA | | AGCCATCGCATCCTTCATGGTTTG | |  |
| Thaumatin-like protein | cassava4.1_011211m.g | | TGACTCCACCCTTGGCATCTTTGA | | TGTGTTTACCGGCACCTCCTCTGT | |  |
| PHE-ammonia lyase(PAL) | cassava4.1_002591m.g | | GCATCTTTGCGTACACTTTGCT | | ACATAAGCCCAAAACTAGCTGGA | |  |
| Cyclin P4 | cassava4.1_016519m.g | | GACAATGGAACAGGAATGGTTAAG | | CTCAAGAGTCCGAGCAACATAG | |  |
| Plant Invertase | cassava4.1_034263m.g | | ACTGTCTCAACCTGTTGCCGGG | | GGCTGCAGCAGAAGCTGACAAAGAA | |  |
| JAZ protein 10 | cassava4.1_016821m.g | | TGCAGAACTGGTCGCCCAAGG | | TGCAGAACTGGTCGCCCAAGG | |  |
| Cytochrome P450 | cassava4.1_029565m.g | | TCAGGCTGACTGTGCTGTTCTCAT | | GACACCAAGGGTGAAAGCAAGCAA | |  |
| Rubisco  methyltransferase | cassava4.1_006869m.g | | GGCAAATAGGAGCCAATGAA | | GGCAAATAGGAGCCAATGAA | |  |
| UBQ10 *  (endogenous control) | DV441403 | | GCGCCAAATTCAAGGACGAGAACT | | ACAGCCTCGGCCTTCTTAATCACA | |  |
| WRKY70 | cassava4.1_013417m.g | | GTCTCACCTACAACAGAAGATGG | | CTTGCACCCTTGATCGTACTT | |  |
| TIR-NBS | cassava4.1_009831m.g | | CTGTTGGACATGGAAGAGAGTT | | AGGATGCACTCCACACATTATTA | |  |
| MAPK3 | cassava4.1_010219m.g | | CGGACTACACTGATGCAATAGAT | | GTGATCGTTGCCTGGAAATAAAG | |  |
| HistoneH3/H4 | cassava4.1_024615m.g | | CAGAGGCTTGTGAGGGAAAT | | CGAAGAGCCCAACAAGGTAA | |  |
| CYC3B | cassava4.1_032656m.g | | GACCAGAGGCCTTTGACTAAT | | CGTATCCGGAACAAGCCTATAC | |  |
| PME3 | cassava4.1_004357m.g | | GCAGGACTGACCCAAATCAA | | TTCCATGGCCTTCCAAGATAAG | |  |
| LOX3 | cassava4.1_001238m.g | | GCCGCTACTGTATGGAGATTAG | | TTGTGTTGGGTCAGGTACAG | |  |
